# Prompt and non-prompt $J/\psi$ and $\psi (2\mathrm {S})$ suppression at high transverse momentum in $5.02~\mathrm {TeV}$ Pb+Pb collisions with the ATLAS experiment

**DOI:** 10.1140/epjc/s10052-018-6219-9

**Published:** 2018-09-21

**Authors:** M. Aaboud, G. Aad, B. Abbott, O. Abdinov, B. Abeloos, S. H. Abidi, O. S. AbouZeid, N. L. Abraham, H. Abramowicz, H. Abreu, Y. Abulaiti, B. S. Acharya, S. Adachi, L. Adamczyk, J. Adelman, M. Adersberger, T. Adye, A. A. Affolder, Y. Afik, C. Agheorghiesei, J. A. Aguilar-Saavedra, F. Ahmadov, G. Aielli, S. Akatsuka, T. P. A. Åkesson, E. Akilli, A. V. Akimov, G. L. Alberghi, J. Albert, P. Albicocco, M. J. Alconada Verzini, S. Alderweireldt, M. Aleksa, I. N. Aleksandrov, C. Alexa, G. Alexander, T. Alexopoulos, M. Alhroob, B. Ali, G. Alimonti, J. Alison, S. P. Alkire, C. Allaire, B. M. M. Allbrooke, B. W. Allen, P. P. Allport, A. Aloisio, A. Alonso, F. Alonso, C. Alpigiani, A. A. Alshehri, M. I. Alstaty, B. Alvarez Gonzalez, D. Álvarez Piqueras, M. G. Alviggi, B. T. Amadio, Y. Amaral Coutinho, L. Ambroz, C. Amelung, D. Amidei, S. P. Amor Dos Santos, S. Amoroso, C. S. Amrouche, C. Anastopoulos, L. S. Ancu, N. Andari, T. Andeen, C. F. Anders, J. K. Anders, K. J. Anderson, A. Andreazza, V. Andrei, S. Angelidakis, I. Angelozzi, A. Angerami, A. V. Anisenkov, A. Annovi, C. Antel, M. T. Anthony, M. Antonelli, D. J. A. Antrim, F. Anulli, M. Aoki, L. Aperio Bella, G. Arabidze, Y. Arai, J. P. Araque, V. Araujo Ferraz, R. Araujo Pereira, A. T. H. Arce, R. E. Ardell, F. A. Arduh, J-F. Arguin, S. Argyropoulos, A. J. Armbruster, L. J. Armitage, O. Arnaez, H. Arnold, M. Arratia, O. Arslan, A. Artamonov, G. Artoni, S. Artz, S. Asai, N. Asbah, A. Ashkenazi, E. M. Asimakopoulou, L. Asquith, K. Assamagan, R. Astalos, R. J. Atkin, M. Atkinson, N. B. Atlay, K. Augsten, G. Avolio, R. Avramidou, B. Axen, M. K. Ayoub, G. Azuelos, A. E. Baas, M. J. Baca, H. Bachacou, K. Bachas, M. Backes, P. Bagnaia, M. Bahmani, H. Bahrasemani, A. J. Bailey, J. T. Baines, M. Bajic, O. K. Baker, P. J. Bakker, D. Bakshi Gupta, E. M. Baldin, P. Balek, F. Balli, W. K. Balunas, E. Banas, A. Bandyopadhyay, S. Banerjee, A. A. E. Bannoura, L. Barak, W. M. Barbe, E. L. Barberio, D. Barberis, M. Barbero, T. Barillari, M-S. Barisits, J. Barkeloo, T. Barklow, N. Barlow, R. Barnea, S. L. Barnes, B. M. Barnett, R. M. Barnett, Z. Barnovska-Blenessy, A. Baroncelli, G. Barone, A. J. Barr, L. Barranco Navarro, F. Barreiro, J. Barreiro Guimarães da Costa, R. Bartoldus, A. E. Barton, P. Bartos, A. Basalaev, A. Bassalat, R. L. Bates, S. J. Batista, S. Batlamous, J. R. Batley, M. Battaglia, M. Bauce, F. Bauer, K. T. Bauer, H. S. Bawa, J. B. Beacham, M. D. Beattie, T. Beau, P. H. Beauchemin, P. Bechtle, H. C. Beck, H. P. Beck, K. Becker, M. Becker, C. Becot, A. Beddall, A. J. Beddall, V. A. Bednyakov, M. Bedognetti, C. P. Bee, T. A. Beermann, M. Begalli, M. Begel, A. Behera, J. K. Behr, A. S. Bell, G. Bella, L. Bellagamba, A. Bellerive, M. Bellomo, K. Belotskiy, N. L. Belyaev, O. Benary, D. Benchekroun, M. Bender, N. Benekos, Y. Benhammou, E. Benhar Noccioli, J. Benitez, D. P. Benjamin, M. Benoit, J. R. Bensinger, S. Bentvelsen, L. Beresford, M. Beretta, D. Berge, E. Bergeaas Kuutmann, N. Berger, L. J. Bergsten, J. Beringer, S. Berlendis, N. R. Bernard, G. Bernardi, C. Bernius, F. U. Bernlochner, T. Berry, P. Berta, C. Bertella, G. Bertoli, I. A. Bertram, C. Bertsche, G. J. Besjes, O. Bessidskaia Bylund, M. Bessner, N. Besson, A. Bethani, S. Bethke, A. Betti, A. J. Bevan, J. Beyer, R. M. B. Bianchi, O. Biebel, D. Biedermann, R. Bielski, K. Bierwagen, N. V. Biesuz, M. Biglietti, T. R. V. Billoud, M. Bindi, A. Bingul, C. Bini, S. Biondi, T. Bisanz, J. P. Biswal, C. Bittrich, D. M. Bjergaard, J. E. Black, K. M. Black, R. E. Blair, T. Blazek, I. Bloch, C. Blocker, A. Blue, U. Blumenschein, Dr. Blunier, G. J. Bobbink, V. S. Bobrovnikov, S. S. Bocchetta, A. Bocci, C. Bock, D. Boerner, D. Bogavac, A. G. Bogdanchikov, C. Bohm, V. Boisvert, P. Bokan, T. Bold, A. S. Boldyrev, A. E. Bolz, M. Bomben, M. Bona, J. S. Bonilla, M. Boonekamp, A. Borisov, G. Borissov, J. Bortfeldt, D. Bortoletto, V. Bortolotto, D. Boscherini, M. Bosman, J. D. Bossio Sola, J. Boudreau, E. V. Bouhova-Thacker, D. Boumediene, C. Bourdarios, S. K. Boutle, A. Boveia, J. Boyd, I. R. Boyko, A. J. Bozson, J. Bracinik, N. Brahimi, A. Brandt, G. Brandt, O. Brandt, F. Braren, U. Bratzler, B. Brau, J. E. Brau, W. D. Breaden Madden, K. Brendlinger, A. J. Brennan, L. Brenner, R. Brenner, S. Bressler, B. Brickwedde, D. L. Briglin, T. M. Bristow, D. Britton, D. Britzger, I. Brock, R. Brock, G. Brooijmans, T. Brooks, W. K. Brooks, E. Brost, J. H Broughton, P. A. Bruckman de Renstrom, D. Bruncko, A. Bruni, G. Bruni, L. S. Bruni, S. Bruno, B. H. Brunt, M. Bruschi, N. Bruscino, P. Bryant, L. Bryngemark, T. Buanes, Q. Buat, P. Buchholz, A. G. Buckley, I. A. Budagov, F. Buehrer, M. K. Bugge, O. Bulekov, D. Bullock, T. J. Burch, S. Burdin, C. D. Burgard, A. M. Burger, B. Burghgrave, K. Burka, S. Burke, I. Burmeister, J. T. P. Burr, D. Büscher, V. Büscher, E. Buschmann, P. Bussey, J. M. Butler, C. M. Buttar, J. M. Butterworth, P. Butti, W. Buttinger, A. Buzatu, A. R. Buzykaev, G. Cabras, S. Cabrera Urbán, D. Caforio, H. Cai, V. M. M. Cairo, O. Cakir, N. Calace, P. Calafiura, A. Calandri, G. Calderini, P. Calfayan, G. Callea, L. P. Caloba, S. Calvente Lopez, D. Calvet, S. Calvet, T. P. Calvet, M. Calvetti, R. Camacho Toro, S. Camarda, P. Camarri, D. Cameron, R. Caminal Armadans, C. Camincher, S. Campana, M. Campanelli, A. Camplani, A. Campoverde, V. Canale, M. Cano Bret, J. Cantero, T. Cao, Y. Cao, M. D. M. Capeans Garrido, I. Caprini, M. Caprini, M. Capua, R. M. Carbone, R. Cardarelli, F. C. Cardillo, I. Carli, T. Carli, G. Carlino, B. T. Carlson, L. Carminati, R. M. D. Carney, S. Caron, E. Carquin, S. Carrá, G. D. Carrillo-Montoya, D. Casadei, M. P. Casado, A. F. Casha, M. Casolino, D. W. Casper, R. Castelijn, V. Castillo Gimenez, N. F. Castro, A. Catinaccio, J. R. Catmore, A. Cattai, J. Caudron, V. Cavaliere, E. Cavallaro, D. Cavalli, M. Cavalli-Sforza, V. Cavasinni, E. Celebi, F. Ceradini, L. Cerda Alberich, A. S. Cerqueira, A. Cerri, L. Cerrito, F. Cerutti, A. Cervelli, S. A. Cetin, A. Chafaq, D. Chakraborty, S. K. Chan, W. S. Chan, Y. L. Chan, P. Chang, J. D. Chapman, D. G. Charlton, C. C. Chau, C. A. Chavez Barajas, S. Che, A. Chegwidden, S. Chekanov, S. V. Chekulaev, G. A. Chelkov, M. A. Chelstowska, C. Chen, C. H. Chen, H. Chen, J. Chen, J. Chen, S. Chen, S. J. Chen, X. Chen, Y. Chen, Y-H. Chen, H. C. Cheng, H. J. Cheng, A. Cheplakov, E. Cheremushkina, R. Cherkaoui El Moursli, E. Cheu, K. Cheung, L. Chevalier, V. Chiarella, G. Chiarelli, G. Chiodini, A. S. Chisholm, A. Chitan, I. Chiu, Y. H. Chiu, M. V. Chizhov, K. Choi, A. R. Chomont, S. Chouridou, Y. S. Chow, V. Christodoulou, M. C. Chu, J. Chudoba, A. J. Chuinard, J. J. Chwastowski, L. Chytka, D. Cinca, V. Cindro, I. A. Cioară, A. Ciocio, F. Cirotto, Z. H. Citron, M. Citterio, A. Clark, M. R. Clark, P. J. Clark, R. N. Clarke, C. Clement, Y. Coadou, M. Cobal, A. Coccaro, J. Cochran, A. E. C. Coimbra, L. Colasurdo, B. Cole, A. P. Colijn, J. Collot, P. Conde Muiño, E. Coniavitis, S. H. Connell, I. A. Connelly, S. Constantinescu, F. Conventi, A. M. Cooper-Sarkar, F. Cormier, K. J. R. Cormier, M. Corradi, E. E. Corrigan, F. Corriveau, A. Cortes-Gonzalez, M. J. Costa, D. Costanzo, G. Cottin, G. Cowan, B. E. Cox, J. Crane, K. Cranmer, S. J. Crawley, R. A. Creager, G. Cree, S. Crépé-Renaudin, F. Crescioli, M. Cristinziani, V. Croft, G. Crosetti, A. Cueto, T. Cuhadar Donszelmann, A. R. Cukierman, M. Curatolo, J. Cúth, S. Czekierda, P. Czodrowski, M. J. Da Cunha Sargedas De Sousa, C. Da Via, W. Dabrowski, T. Dado, S. Dahbi, T. Dai, O. Dale, F. Dallaire, C. Dallapiccola, M. Dam, G. D’amen, J. R. Dandoy, M. F. Daneri, N. P. Dang, N. D. Dann, M. Danninger, V. Dao, G. Darbo, S. Darmora, O. Dartsi, A. Dattagupta, T. Daubney, S. D’Auria, W. Davey, C. David, T. Davidek, D. R. Davis, E. Dawe, I. Dawson, K. De, R. De Asmundis, A. De Benedetti, S. De Castro, S. De Cecco, N. De Groot, P. de Jong, H. De la Torre, F. De Lorenzi, A. De Maria, D. De Pedis, A. De Salvo, U. De Sanctis, A. De Santo, K. De Vasconcelos Corga, J. B. De Vivie De Regie, C. Debenedetti, D. V. Dedovich, N. Dehghanian, M. Del Gaudio, J. Del Peso, D. Delgove, F. Deliot, C. M. Delitzsch, M. Della Pietra, D. Della Volpe, A. Dell’Acqua, L. Dell’Asta, M. Delmastro, C. Delporte, P. A. Delsart, D. A. DeMarco, S. Demers, M. Demichev, S. P. Denisov, D. Denysiuk, L. D’Eramo, D. Derendarz, J. E. Derkaoui, F. Derue, P. Dervan, K. Desch, C. Deterre, K. Dette, M. R. Devesa, P. O. Deviveiros, A. Dewhurst, S. Dhaliwal, F. A. Di Bello, A. Di Ciaccio, L. Di Ciaccio, W. K. Di Clemente, C. Di Donato, A. Di Girolamo, B. Di Micco, R. Di Nardo, K. F. Di Petrillo, A. Di Simone, R. Di Sipio, D. Di Valentino, C. Diaconu, M. Diamond, F. A. Dias, T. Dias Do Vale, M. A. Diaz, J. Dickinson, E. B. Diehl, J. Dietrich, S. Díez Cornell, A. Dimitrievska, J. Dingfelder, F. Dittus, F. Djama, T. Djobava, J. I. Djuvsland, M. A. B. Do Vale, M. Dobre, D. Dodsworth, C. Doglioni, J. Dolejsi, Z. Dolezal, M. Donadelli, J. Donini, A. D’onofrio, M. D’Onofrio, J. Dopke, A. Doria, M. T. Dova, A. T. Doyle, E. Drechsler, E. Dreyer, T. Dreyer, M. Dris, Y. Du, J. Duarte-Campderros, F. Dubinin, A. Dubreuil, E. Duchovni, G. Duckeck, A. Ducourthial, O. A. Ducu, D. Duda, A. Dudarev, A. C. Dudder, E. M. Duffield, L. Duflot, M. Dührssen, C. Dülsen, M. Dumancic, A. E. Dumitriu, A. K. Duncan, M. Dunford, A. Duperrin, H. DuranYildiz, M. Düren, A. Durglishvili, D. Duschinger, B. Dutta, D. Duvnjak, M. Dyndal, B. S. Dziedzic, C. Eckardt, K. M. Ecker, R. C. Edgar, T. Eifert, G. Eigen, K. Einsweiler, T. Ekelof, M. El Kacimi, R. El Kosseifi, V. Ellajosyula, M. Ellert, F. Ellinghaus, A. A. Elliot, N. Ellis, J. Elmsheuser, M. Elsing, D. Emeliyanov, Y. Enari, J. S. Ennis, M. B. Epland, J. Erdmann, A. Ereditato, S. Errede, M. Escalier, C. Escobar, B. Esposito, O. EstradaPastor, A. I. Etienvre, E. Etzion, H. Evans, A. Ezhilov, M. Ezzi, F. Fabbri, L. Fabbri, V. Fabiani, G. Facini, R. M. Faisca Rodrigues Pereira, R. M. Fakhrutdinov, S. Falciano, P. J. Falke, S. Falke, J. Faltova, Y. Fang, M. Fanti, A. Farbin, A. Farilla, E. M. Farina, T. Farooque, S. Farrell, S. M. Farrington, P. Farthouat, F. Fassi, P. Fassnacht, D. Fassouliotis, M. Faucci Giannelli, A. Favareto, W. J. Fawcett, L. Fayard, O. L. Fedin, W. Fedorko, M. Feickert, S. Feigl, L. Feligioni, C. Feng, E. J. Feng, M. Feng, M. J. Fenton, A. B. Fenyuk, L. Feremenga, J. Ferrando, A. Ferrari, P. Ferrari, R. Ferrari, D. E. Ferreira de Lima, A. Ferrer, D. Ferrere, C. Ferretti, F. Fiedler, A. Filipčič, F. Filthaut, M. Fincke-Keeler, K. D. Finelli, M. C. N. Fiolhais, L. Fiorini, C. Fischer, J. Fischer, W. C. Fisher, N. Flaschel, I. Fleck, P. Fleischmann, R. R. M. Fletcher, T. Flick, B. M. Flierl, L. M. Flores, L. R. Flores Castillo, N. Fomin, G. T. Forcolin, A. Formica, F. A. Förster, A. C. Forti, A. G. Foster, D. Fournier, H. Fox, S. Fracchia, P. Francavilla, M. Franchini, S. Franchino, D. Francis, L. Franconi, M. Franklin, M. Frate, M. Fraternali, D. Freeborn, S. M. Fressard-Batraneanu, B. Freund, W. S. Freund, D. Froidevaux, J. A. Frost, C. Fukunaga, T. Fusayasu, J. Fuster, O. Gabizon, A. Gabrielli, A. Gabrielli, G. P. Gach, S. Gadatsch, S. Gadomski, P. Gadow, G. Gagliardi, L. G. Gagnon, C. Galea, B. Galhardo, E. J. Gallas, B. J. Gallop, P. Gallus, G. Galster, R. Gamboa Goni, K. K. Gan, S. Ganguly, Y. Gao, Y. S. Gao, C. García, J. E. García Navarro, J. A. García Pascual, M. Garcia-Sciveres, R. W. Gardner, N. Garelli, V. Garonne, K. Gasnikova, A. Gaudiello, G. Gaudio, I. L. Gavrilenko, A. Gavrilyuk, C. Gay, G. Gaycken, E. N. Gazis, C. N. P. Gee, J. Geisen, M. Geisen, M. P. Geisler, K. Gellerstedt, C. Gemme, M. H. Genest, C. Geng, S. Gentile, C. Gentsos, S. George, D. Gerbaudo, G. Gessner, S. Ghasemi, M. Ghneimat, B. Giacobbe, S. Giagu, N. Giangiacomi, P. Giannetti, S. M. Gibson, M. Gignac, D. Gillberg, G. Gilles, D. M. Gingrich, M. P. Giordani, F. M. Giorgi, P. F. Giraud, P. Giromini, G. Giugliarelli, D. Giugni, F. Giuli, M. Giulini, S. Gkaitatzis, I. Gkialas, E. L. Gkougkousis, P. Gkountoumis, L. K. Gladilin, C. Glasman, J. Glatzer, P. C. F. Glaysher, A. Glazov, M. Goblirsch-Kolb, J. Godlewski, S. Goldfarb, T. Golling, D. Golubkov, A. Gomes, R. Goncalves Gama, R. Gonçalo, G. Gonella, L. Gonella, A. Gongadze, F. Gonnella, J. L. Gonski, S. González de la Hoz, S. Gonzalez-Sevilla, L. Goossens, P. A. Gorbounov, H. A. Gordon, B. Gorini, E. Gorini, A. Gorišek, A. T. Goshaw, C. Gössling, M. I. Gostkin, C. A. Gottardo, C. R. Goudet, D. Goujdami, A. G. Goussiou, N. Govender, C. Goy, E. Gozani, I. Grabowska-Bold, P. O. J. Gradin, E. C. Graham, J. Gramling, E. Gramstad, S. Grancagnolo, V. Gratchev, P. M. Gravila, C. Gray, H. M. Gray, Z. D. Greenwood, C. Grefe, K. Gregersen, I. M. Gregor, P. Grenier, K. Grevtsov, J. Griffiths, A. A. Grillo, K. Grimm, S. Grinstein, Ph. Gris, J.-F. Grivaz, S. Groh, E. Gross, J. Grosse-Knetter, G. C. Grossi, Z. J. Grout, A. Grummer, L. Guan, W. Guan, J. Guenther, A. Guerguichon, F. Guescini, D. Guest, O. Gueta, R. Gugel, B. Gui, T. Guillemin, S. Guindon, U. Gul, C. Gumpert, J. Guo, W. Guo, Y. Guo, Z. Guo, R. Gupta, S. Gurbuz, G. Gustavino, B. J. Gutelman, P. Gutierrez, N. G. Gutierrez Ortiz, C. Gutschow, C. Guyot, M. P. Guzik, C. Gwenlan, C. B. Gwilliam, A. Haas, C. Haber, H. K. Hadavand, N. Haddad, A. Hadef, S. Hageböck, M. Hagihara, H. Hakobyan, M. Haleem, J. Haley, G. Halladjian, G. D. Hallewell, K. Hamacher, P. Hamal, K. Hamano, A. Hamilton, G. N. Hamity, K. Han, L. Han, S. Han, K. Hanagaki, M. Hance, D. M. Handl, B. Haney, R. Hankache, P. Hanke, E. Hansen, J. B. Hansen, J. D. Hansen, M. C. Hansen, P. H. Hansen, K. Hara, A. S. Hard, T. Harenberg, S. Harkusha, P. F. Harrison, N. M. Hartmann, Y. Hasegawa, A. Hasib, S. Hassani, S. Haug, R. Hauser, L. Hauswald, L. B. Havener, M. Havranek, C. M. Hawkes, R. J. Hawkings, D. Hayden, C. Hayes, C. P. Hays, J. M. Hays, H. S. Hayward, S. J. Haywood, M. P. Heath, V. Hedberg, L. Heelan, S. Heer, K. K. Heidegger, J. Heilman, S. Heim, T. Heim, B. Heinemann, J. J. Heinrich, L. Heinrich, C. Heinz, J. Hejbal, L. Helary, A. Held, S. Hellesund, S. Hellman, C. Helsens, R. C. W. Henderson, Y. Heng, S. Henkelmann, A. M. Henriques Correia, G. H. Herbert, H. Herde, V. Herget, Y. Hernández Jiménez, H. Herr, G. Herten, R. Hertenberger, L. Hervas, T. C. Herwig, G. G. Hesketh, N. P. Hessey, J. W. Hetherly, S. Higashino, E. Higón-Rodriguez, K. Hildebrand, E. Hill, J. C. Hill, K. H. Hiller, S. J. Hillier, M. Hils, I. Hinchliffe, M. Hirose, D. Hirschbuehl, B. Hiti, O. Hladik, D. R. Hlaluku, X. Hoad, J. Hobbs, N. Hod, M. C. Hodgkinson, A. Hoecker, M. R. Hoeferkamp, F. Hoenig, D. Hohn, D. Hohov, T. R. Holmes, M. Holzbock, M. Homann, S. Honda, T. Honda, T. M. Hong, A. Hönle, B. H. Hooberman, W. H. Hopkins, Y. Horii, P. Horn, A. J. Horton, L. A. Horyn, J-Y. Hostachy, A. Hostiuc, S. Hou, A. Hoummada, J. Howarth, J. Hoya, M. Hrabovsky, J. Hrdinka, I. Hristova, J. Hrivnac, A. Hrynevich, T. Hryn’ova, P. J. Hsu, S.-C. Hsu, Q. Hu, S. Hu, Y. Huang, Z. Hubacek, F. Hubaut, M. Huebner, F. Huegging, T. B. Huffman, E. W. Hughes, M. Huhtinen, R. F. H. Hunter, P. Huo, A. M. Hupe, N. Huseynov, J. Huston, J. Huth, R. Hyneman, G. Iacobucci, G. Iakovidis, I. Ibragimov, L. Iconomidou-Fayard, Z. Idrissi, P. Iengo, R. Ignazzi, O. Igonkina, R. Iguchi, T. Iizawa, Y. Ikegami, M. Ikeno, D. Iliadis, N. Ilic, F. Iltzsche, G. Introzzi, M. Iodice, K. Iordanidou, V. Ippolito, M. F. Isacson, N. Ishijima, M. Ishino, M. Ishitsuka, C. Issever, S. Istin, F. Ito, J. M. Iturbe Ponce, R. Iuppa, A. Ivina, H. Iwasaki, J. M. Izen, V. Izzo, S. Jabbar, P. Jacka, P. Jackson, R. M. Jacobs, V. Jain, G. Jäkel, K. B. Jakobi, K. Jakobs, S. Jakobsen, T. Jakoubek, D. O. Jamin, D. K. Jana, R. Jansky, J. Janssen, M. Janus, P. A. Janus, G. Jarlskog, N. Javadov, T. Javůrek, M. Javurkova, F. Jeanneau, L. Jeanty, J. Jejelava, A. Jelinskas, P. Jenni, J. Jeong, C. Jeske, S. Jézéquel, H. Ji, J. Jia, H. Jiang, Y. Jiang, Z. Jiang, S. Jiggins, F. A. Jimenez Morales, J. Jimenez Pena, S. Jin, A. Jinaru, O. Jinnouchi, H. Jivan, P. Johansson, K. A. Johns, C. A. Johnson, W. J. Johnson, K. Jon-And, R. W. L. Jones, S. D. Jones, S. Jones, T. J. Jones, J. Jongmanns, P. M. Jorge, J. Jovicevic, X. Ju, J. J. Junggeburth, A. Juste Rozas, A. Kaczmarska, M. Kado, H. Kagan, M. Kagan, T. Kaji, E. Kajomovitz, C. W. Kalderon, A. Kaluza, S. Kama, A. Kamenshchikov, L. Kanjir, Y. Kano, V. A. Kantserov, J. Kanzaki, B. Kaplan, L. S. Kaplan, D. Kar, M. J. Kareem, E. Karentzos, S. N. Karpov, Z. M. Karpova, V. Kartvelishvili, A. N. Karyukhin, K. Kasahara, L. Kashif, R. D. Kass, A. Kastanas, Y. Kataoka, C. Kato, A. Katre, J. Katzy, K. Kawade, K. Kawagoe, T. Kawamoto, G. Kawamura, E. F. Kay, V. F. Kazanin, R. Keeler, R. Kehoe, J. S. Keller, E. Kellermann, J. J. Kempster, J. Kendrick, O. Kepka, S. Kersten, B. P. Kerševan, R. A. Keyes, M. Khader, F. Khalil-Zada, A. Khanov, A. G. Kharlamov, T. Kharlamova, A. Khodinov, T. J. Khoo, V. Khovanskiy, E. Khramov, J. Khubua, S. Kido, M. Kiehn, C. R. Kilby, H. Y. Kim, S. H. Kim, Y. K. Kim, N. Kimura, O. M. Kind, B. T. King, D. Kirchmeier, J. Kirk, A. E. Kiryunin, T. Kishimoto, D. Kisielewska, V. Kitali, O. Kivernyk, E. Kladiva, T. Klapdor-Kleingrothaus, M. H. Klein, M. Klein, U. Klein, K. Kleinknecht, P. Klimek, A. Klimentov, R. Klingenberg, T. Klingl, T. Klioutchnikova, F. F. Klitzner, P. Kluit, S. Kluth, E. Kneringer, E. B. F. G. Knoops, A. Knue, A. Kobayashi, D. Kobayashi, T. Kobayashi, M. Kobel, M. Kocian, P. Kodys, T. Koffas, E. Koffeman, N. M. Köhler, T. Koi, M. Kolb, I. Koletsou, T. Kondo, N. Kondrashova, K. Köneke, A. C. König, T. Kono, R. Konoplich, N. Konstantinidis, B. Konya, R. Kopeliansky, S. Koperny, K. Korcyl, K. Kordas, A. Korn, I. Korolkov, E. V. Korolkova, O. Kortner, S. Kortner, T. Kosek, V. V. Kostyukhin, A. Kotwal, A. Koulouris, A. Kourkoumeli-Charalampidi, C. Kourkoumelis, E. Kourlitis, V. Kouskoura, A. B. Kowalewska, R. Kowalewski, T. Z. Kowalski, C. Kozakai, W. Kozanecki, A. S. Kozhin, V. A. Kramarenko, G. Kramberger, D. Krasnopevtsev, M. W. Krasny, A. Krasznahorkay, D. Krauss, J. A. Kremer, J. Kretzschmar, K. Kreutzfeldt, P. Krieger, K. Krizka, K. Kroeninger, H. Kroha, J. Kroll, J. Kroll, J. Kroseberg, J. Krstic, U. Kruchonak, H. Krüger, N. Krumnack, M. C. Kruse, T. Kubota, S. Kuday, J. T. Kuechler, S. Kuehn, A. Kugel, F. Kuger, T. Kuhl, V. Kukhtin, R. Kukla, Y. Kulchitsky, S. Kuleshov, Y. P. Kulinich, M. Kuna, T. Kunigo, A. Kupco, T. Kupfer, O. Kuprash, H. Kurashige, L. L. Kurchaninov, Y. A. Kurochkin, M. G. Kurth, E. S. Kuwertz, M. Kuze, J. Kvita, T. Kwan, A. La Rosa, J. L. La Rosa Navarro, L. La Rotonda, F. La Ruffa, C. Lacasta, F. Lacava, J. Lacey, D. P. J. Lack, H. Lacker, D. Lacour, E. Ladygin, R. Lafaye, B. Laforge, S. Lai, S. Lammers, W. Lampl, E. Lançon, U. Landgraf, M. P. J. Landon, M. C. Lanfermann, V. S. Lang, J. C. Lange, R. J. Langenberg, A. J. Lankford, F. Lanni, K. Lantzsch, A. Lanza, A. Lapertosa, S. Laplace, J. F. Laporte, T. Lari, F. Lasagni Manghi, M. Lassnig, T. S. Lau, A. Laudrain, A. T. Law, P. Laycock, M. Lazzaroni, B. Le, O. Le Dortz, E. Le Guirriec, E. P. Le Quilleuc, M. LeBlanc, T. LeCompte, F. Ledroit-Guillon, C. A. Lee, G. R. Lee, L. Lee, S. C. Lee, B. Lefebvre, M. Lefebvre, F. Legger, C. Leggett, G. Lehmann Miotto, W. A. Leight, A. Leisos, M. A. L. Leite, R. Leitner, D. Lellouch, B. Lemmer, K. J. C. Leney, T. Lenz, B. Lenzi, R. Leone, S. Leone, C. Leonidopoulos, G. Lerner, C. Leroy, R. Les, A. A. J. Lesage, C. G. Lester, M. Levchenko, J. Levêque, D. Levin, L. J. Levinson, D. Lewis, B. Li, C-Q. Li, H. Li, L. Li, Q. Li, Q. Y. Li, S. Li, X. Li, Y. Li, Z. Liang, B. Liberti, A. Liblong, K. Lie, S. Liem, A. Limosani, C. Y. Lin, K. Lin, S. C. Lin, T. H. Lin, R. A. Linck, B. E. Lindquist, A. L. Lionti, E. Lipeles, A. Lipniacka, M. Lisovyi, T. M. Liss, A. Lister, A. M. Litke, J. D. Little, B. Liu, B. L. Liu, H. B. Liu, H. Liu, J. B. Liu, J. K. K. Liu, K. Liu, M. Liu, P. Liu, Y. L. Liu, Y. W. Liu, M. Livan, A. Lleres, J. Llorente Merino, S. L. Lloyd, C. Y. Lo, F. Lo Sterzo, E. M. Lobodzinska, P. Loch, F. K. Loebinger, A. Loesle, K. M. Loew, T. Lohse, K. Lohwasser, M. Lokajicek, B. A. Long, J. D. Long, R. E. Long, L. Longo, K. A. Looper, J. A. Lopez, I. Lopez Paz, A. Lopez Solis, J. Lorenz, N. Lorenzo Martinez, M. Losada, P. J. Lösel, X. Lou, X. Lou, A. Lounis, J. Love, P. A. Love, J. J. Lozano Bahilo, H. Lu, N. Lu, Y. J. Lu, H. J. Lubatti, C. Luci, A. Lucotte, C. Luedtke, F. Luehring, I. Luise, W. Lukas, L. Luminari, B. Lund-Jensen, M. S. Lutz, P. M. Luzi, D. Lynn, R. Lysak, E. Lytken, F. Lyu, V. Lyubushkin, H. Ma, L. L. Ma, Y. Ma, G. Maccarrone, A. Macchiolo, C. M. Macdonald, J. Machado Miguens, D. Madaffari, R. Madar, W. F. Mader, A. Madsen, N. Madysa, J. Maeda, S. Maeland, T. Maeno, A. S. Maevskiy, V. Magerl, C. Maidantchik, T. Maier, A. Maio, O. Majersky, S. Majewski, Y. Makida, N. Makovec, B. Malaescu, Pa. Malecki, V. P. Maleev, F. Malek, U. Mallik, D. Malon, C. Malone, S. Maltezos, S. Malyukov, J. Mamuzic, G. Mancini, I. Mandić, J. Maneira, L. Manhaes de Andrade Filho, J. Manjarres Ramos, K. H. Mankinen, A. Mann, A. Manousos, B. Mansoulie, J. D. Mansour, R. Mantifel, M. Mantoani, S. Manzoni, G. Marceca, L. March, L. Marchese, G. Marchiori, M. Marcisovsky, C. A. Marin Tobon, M. Marjanovic, D. E. Marley, F. Marroquim, Z. Marshall, M. U. F Martensson, S. Marti-Garcia, C. B. Martin, T. A. Martin, V. J. Martin, B. Martin dit Latour, M. Martinez, V. I. Martinez Outschoorn, S. Martin-Haugh, V. S. Martoiu, A. C. Martyniuk, A. Marzin, L. Masetti, T. Mashimo, R. Mashinistov, J. Masik, A. L. Maslennikov, L. H. Mason, L. Massa, P. Mastrandrea, A. Mastroberardino, T. Masubuchi, P. Mättig, J. Maurer, B. Maček, S. J. Maxfield, D. A. Maximov, R. Mazini, I. Maznas, S. M. Mazza, N. C. Mc Fadden, G. Mc Goldrick, S. P. Mc Kee, A. McCarn, T. G. McCarthy, L. I. McClymont, E. F. McDonald, J. A. Mcfayden, G. Mchedlidze, M. A. McKay, K. D. McLean, S. J. McMahon, P. C. McNamara, C. J. McNicol, R. A. McPherson, J. E. Mdhluli, Z. A. Meadows, S. Meehan, T. Megy, S. Mehlhase, A. Mehta, T. Meideck, B. Meirose, D. Melini, B. R. Mellado Garcia, J. D. Mellenthin, M. Melo, F. Meloni, A. Melzer, S. B. Menary, L. Meng, X. T. Meng, A. Mengarelli, S. Menke, E. Meoni, S. Mergelmeyer, C. Merlassino, P. Mermod, L. Merola, C. Meroni, F. S. Merritt, A. Messina, J. Metcalfe, A. S. Mete, C. Meyer, J. Meyer, J-P. Meyer, H. Meyer Zu Theenhausen, F. Miano, R. P. Middleton, L. Mijović, G. Mikenberg, M. Mikestikova, M. Mikuž, M. Milesi, A. Milic, D. A. Millar, D. W. Miller, A. Milov, D. A. Milstead, A. A. Minaenko, I. A. Minashvili, A. I. Mincer, B. Mindur, M. Mineev, Y. Minegishi, Y. Ming, L. M. Mir, A. Mirto, K. P. Mistry, T. Mitani, J. Mitrevski, V. A. Mitsou, A. Miucci, P. S. Miyagawa, A. Mizukami, J. U. Mjörnmark, T. Mkrtchyan, M. Mlynarikova, T. Moa, K. Mochizuki, P. Mogg, S. Mohapatra, S. Molander, R. Moles-Valls, M. C. Mondragon, K. Mönig, J. Monk, E. Monnier, A. Montalbano, J. Montejo Berlingen, F. Monticelli, S. Monzani, R. W. Moore, N. Morange, D. Moreno, M. Moreno Llácer, P. Morettini, M. Morgenstern, S. Morgenstern, D. Mori, T. Mori, M. Morii, M. Morinaga, V. Morisbak, A. K. Morley, G. Mornacchi, J. D. Morris, L. Morvaj, P. Moschovakos, M. Mosidze, H. J. Moss, J. Moss, K. Motohashi, R. Mount, E. Mountricha, E. J. W. Moyse, S. Muanza, F. Mueller, J. Mueller, R. S. P. Mueller, D. Muenstermann, P. Mullen, G. A. Mullier, F. J. Munoz Sanchez, P. Murin, W. J. Murray, A. Murrone, M. Muškinja, C. Mwewa, A. G. Myagkov, J. Myers, M. Myska, B. P. Nachman, O. Nackenhorst, K. Nagai, R. Nagai, K. Nagano, Y. Nagasaka, K. Nagata, M. Nagel, E. Nagy, A. M. Nairz, Y. Nakahama, K. Nakamura, T. Nakamura, I. Nakano, F. Napolitano, R. F. Naranjo Garcia, R. Narayan, D. I. Narrias Villar, I. Naryshkin, T. Naumann, G. Navarro, R. Nayyar, H. A. Neal, P. Y. Nechaeva, T. J. Neep, A. Negri, M. Negrini, S. Nektarijevic, C. Nellist, M. E. Nelson, S. Nemecek, P. Nemethy, M. Nessi, M. S. Neubauer, M. Neumann, P. R. Newman, T. Y. Ng, Y. S. Ng, H. D. N. Nguyen, T. Nguyen Manh, E. Nibigira, R. B. Nickerson, R. Nicolaidou, J. Nielsen, N. Nikiforou, V. Nikolaenko, I. Nikolic-Audit, K. Nikolopoulos, P. Nilsson, Y. Ninomiya, A. Nisati, N. Nishu, R. Nisius, I. Nitsche, T. Nitta, T. Nobe, Y. Noguchi, M. Nomachi, I. Nomidis, M. A. Nomura, T. Nooney, M. Nordberg, N. Norjoharuddeen, T. Novak, O. Novgorodova, R. Novotny, M. Nozaki, L. Nozka, K. Ntekas, E. Nurse, F. Nuti, F. G. Oakham, H. Oberlack, T. Obermann, J. Ocariz, A. Ochi, I. Ochoa, J. P. Ochoa-Ricoux, K. O’Connor, S. Oda, S. Odaka, A. Oh, S. H. Oh, C. C. Ohm, H. Ohman, H. Oide, H. Okawa, Y. Okazaki, Y. Okumura, T. Okuyama, A. Olariu, L. F. Oleiro Seabra, S. A. Olivares Pino, D. Oliveira Damazio, J. L. Oliver, M. J. R. Olsson, A. Olszewski, J. Olszowska, D. C. O’Neil, A. Onofre, K. Onogi, P. U. E. Onyisi, H. Oppen, M. J. Oreglia, Y. Oren, D. Orestano, E. C. Orgill, N. Orlando, A. A. O’Rourke, R. S. Orr, B. Osculati, V. O’Shea, R. Ospanov, G. Otero y Garzon, H. Otono, M. Ouchrif, F. Ould-Saada, A. Ouraou, Q. Ouyang, M. Owen, R. E. Owen, V. E. Ozcan, N. Ozturk, K. Pachal, A. Pacheco Pages, L. Pacheco Rodriguez, C. Padilla Aranda, S. Pagan Griso, M. Paganini, G. Palacino, S. Palazzo, S. Palestini, M. Palka, D. Pallin, I. Panagoulias, C. E. Pandini, J. G. Panduro Vazquez, P. Pani, L. Paolozzi, T. D. Papadopoulou, K. Papageorgiou, A. Paramonov, D. Paredes Hernandez, B. Parida, A. J. Parker, K. A. Parker, M. A. Parker, F. Parodi, J. A. Parsons, U. Parzefall, V. R. Pascuzzi, J. M. P. Pasner, E. Pasqualucci, S. Passaggio, F. Pastore, P. Pasuwan, S. Pataraia, J. R. Pater, A. Pathak, T. Pauly, B. Pearson, M. Pedersen, S. Pedraza Lopez, R. Pedro, S. V. Peleganchuk, O. Penc, C. Peng, H. Peng, J. Penwell, B. S. Peralva, M. M. Perego, A. P. Pereira Peixoto, D. V. Perepelitsa, F. Peri, L. Perini, H. Pernegger, S. Perrella, V. D. Peshekhonov, K. Peters, R. F. Y. Peters, B. A. Petersen, T. C. Petersen, E. Petit, A. Petridis, C. Petridou, P. Petroff, E. Petrolo, M. Petrov, F. Petrucci, N. E. Pettersson, A. Peyaud, R. Pezoa, T. Pham, F. H. Phillips, P. W. Phillips, G. Piacquadio, E. Pianori, A. Picazio, M. A. Pickering, R. Piegaia, J. E. Pilcher, A. D. Pilkington, M. Pinamonti, J. L. Pinfold, M. Pitt, M-A. Pleier, V. Pleskot, E. Plotnikova, D. Pluth, P. Podberezko, R. Poettgen, R. Poggi, L. Poggioli, I. Pogrebnyak, D. Pohl, I. Pokharel, G. Polesello, A. Poley, A. Policicchio, R. Polifka, A. Polini, C. S. Pollard, V. Polychronakos, D. Ponomarenko, L. Pontecorvo, G. A. Popeneciu, D. M. Portillo Quintero, S. Pospisil, K. Potamianos, I. N. Potrap, C. J. Potter, H. Potti, T. Poulsen, J. Poveda, T. D. Powell, M. E. Pozo Astigarraga, P. Pralavorio, S. Prell, D. Price, M. Primavera, S. Prince, N. Proklova, K. Prokofiev, F. Prokoshin, S. Protopopescu, J. Proudfoot, M. Przybycien, A. Puri, P. Puzo, J. Qian, Y. Qin, A. Quadt, M. Queitsch-Maitland, A. Qureshi, S. K. Radhakrishnan, P. Rados, F. Ragusa, G. Rahal, J. A. Raine, S. Rajagopalan, T. Rashid, S. Raspopov, M. G. Ratti, D. M. Rauch, F. Rauscher, S. Rave, B. Ravina, I. Ravinovich, J. H. Rawling, M. Raymond, A. L. Read, N. P. Readioff, M. Reale, D. M. Rebuzzi, A. Redelbach, G. Redlinger, R. Reece, R. G. Reed, K. Reeves, L. Rehnisch, J. Reichert, A. Reiss, C. Rembser, H. Ren, M. Rescigno, S. Resconi, E. D. Resseguie, S. Rettie, E. Reynolds, O. L. Rezanova, P. Reznicek, R. Richter, S. Richter, E. Richter-Was, O. Ricken, M. Ridel, P. Rieck, C. J. Riegel, O. Rifki, M. Rijssenbeek, A. Rimoldi, M. Rimoldi, L. Rinaldi, G. Ripellino, B. Ristić, E. Ritsch, I. Riu, J. C. Rivera Vergara, F. Rizatdinova, E. Rizvi, C. Rizzi, R. T. Roberts, S. H. Robertson, A. Robichaud-Veronneau, D. Robinson, J. E. M. Robinson, A. Robson, E. Rocco, C. Roda, Y. Rodina, S. Rodriguez Bosca, A. Rodriguez Perez, D. Rodriguez Rodriguez, A. M. Rodríguez Vera, S. Roe, C. S. Rogan, O. Røhne, R. Röhrig, C. P. A. Roland, J. Roloff, A. Romaniouk, M. Romano, E. Romero Adam, N. Rompotis, M. Ronzani, L. Roos, S. Rosati, K. Rosbach, P. Rose, N-A. Rosien, E. Rossi, L. P. Rossi, L. Rossini, J. H. N. Rosten, R. Rosten, M. Rotaru, J. Rothberg, D. Rousseau, D. Roy, A. Rozanov, Y. Rozen, X. Ruan, F. Rubbo, F. Rühr, A. Ruiz-Martinez, Z. Rurikova, N. A. Rusakovich, H. L. Russell, J. P. Rutherfoord, N. Ruthmann, E. M. Rüttinger, Y. F. Ryabov, M. Rybar, G. Rybkin, S. Ryu, A. Ryzhov, G. F. Rzehorz, P. Sabatini, G. Sabato, S. Sacerdoti, H. F-W. Sadrozinski, R. Sadykov, F. Safai Tehrani, P. Saha, M. Sahinsoy, M. Saimpert, M. Saito, T. Saito, H. Sakamoto, A. Sakharov, D. Salamani, G. Salamanna, J. E. Salazar Loyola, D. Salek, P. H. Sales De Bruin, D. Salihagic, A. Salnikov, J. Salt, D. Salvatore, F. Salvatore, A. Salvucci, A. Salzburger, D. Sammel, D. Sampsonidis, D. Sampsonidou, J. Sánchez, A. Sanchez Pineda, H. Sandaker, C. O. Sander, M. Sandhoff, C. Sandoval, D. P. C. Sankey, M. Sannino, Y. Sano, A. Sansoni, C. Santoni, H. Santos, I. Santoyo Castillo, A. Sapronov, J. G. Saraiva, O. Sasaki, K. Sato, E. Sauvan, P. Savard, N. Savic, R. Sawada, C. Sawyer, L. Sawyer, C. Sbarra, A. Sbrizzi, T. Scanlon, D. A. Scannicchio, J. Schaarschmidt, P. Schacht, B. M. Schachtner, D. Schaefer, L. Schaefer, J. Schaeffer, S. Schaepe, U. Schäfer, A. C. Schaffer, D. Schaile, R. D. Schamberger, N. Scharmberg, V. A. Schegelsky, D. Scheirich, F. Schenck, M. Schernau, C. Schiavi, S. Schier, L. K. Schildgen, Z. M. Schillaci, E. J. Schioppa, M. Schioppa, K. E. Schleicher, S. Schlenker, K. R. Schmidt-Sommerfeld, K. Schmieden, C. Schmitt, S. Schmitt, S. Schmitz, U. Schnoor, L. Schoeffel, A. Schoening, E. Schopf, M. Schott, J. F. P. Schouwenberg, J. Schovancova, S. Schramm, N. Schuh, A. Schulte, H-C. Schultz-Coulon, M. Schumacher, B. A. Schumm, Ph. Schune, A. Schwartzman, T. A. Schwarz, H. Schweiger, Ph. Schwemling, R. Schwienhorst, A. Sciandra, G. Sciolla, M. Scornajenghi, F. Scuri, F. Scutti, L. M. Scyboz, J. Searcy, C. D. Sebastiani, P. Seema, S. C. Seidel, A. Seiden, J. M. Seixas, G. Sekhniaidze, K. Sekhon, S. J. Sekula, N. Semprini-Cesari, S. Senkin, C. Serfon, L. Serin, L. Serkin, M. Sessa, H. Severini, F. Sforza, A. Sfyrla, E. Shabalina, J. D. Shahinian, N. W. Shaikh, L. Y. Shan, R. Shang, J. T. Shank, M. Shapiro, A. S. Sharma, A. Sharma, P. B. Shatalov, K. Shaw, S. M. Shaw, A. Shcherbakova, C. Y. Shehu, Y. Shen, N. Sherafati, A. D. Sherman, P. Sherwood, L. Shi, S. Shimizu, C. O. Shimmin, M. Shimojima, I. P. J. Shipsey, S. Shirabe, M. Shiyakova, J. Shlomi, A. Shmeleva, D. Shoaleh Saadi, M. J. Shochet, S. Shojaii, D. R. Shope, S. Shrestha, E. Shulga, P. Sicho, A. M. Sickles, P. E. Sidebo, E. Sideras Haddad, O. Sidiropoulou, A. Sidoti, F. Siegert, Dj. Sijacki, J. Silva, M. Silva, S. B. Silverstein, L. Simic, S. Simion, E. Simioni, B. Simmons, M. Simon, P. Sinervo, N. B. Sinev, M. Sioli, G. Siragusa, I. Siral, S. Yu. Sivoklokov, J. Sjölin, M. B. Skinner, P. Skubic, M. Slater, T. Slavicek, M. Slawinska, K. Sliwa, R. Slovak, V. Smakhtin, B. H. Smart, J. Smiesko, N. Smirnov, S. Yu. Smirnov, Y. Smirnov, L. N. Smirnova, O. Smirnova, J. W. Smith, M. N. K. Smith, R. W. Smith, M. Smizanska, K. Smolek, A. A. Snesarev, I. M. Snyder, S. Snyder, R. Sobie, F. Socher, A. M. Soffa, A. Soffer, A. Søgaard, D. A. Soh, G. Sokhrannyi, C. A. Solans Sanchez, M. Solar, E. Yu. Soldatov, U. Soldevila, A. A. Solodkov, A. Soloshenko, O. V. Solovyanov, V. Solovyev, P. Sommer, H. Son, W. Song, A. Sopczak, F. Sopkova, D. Sosa, C. L. Sotiropoulou, S. Sottocornola, R. Soualah, A. M. Soukharev, D. South, B. C. Sowden, S. Spagnolo, M. Spalla, M. Spangenberg, F. Spanò, D. Sperlich, F. Spettel, T. M. Spieker, R. Spighi, G. Spigo, L. A. Spiller, M. Spousta, A. Stabile, R. Stamen, S. Stamm, E. Stanecka, R. W. Stanek, C. Stanescu, M. M. Stanitzki, B. S. Stapf, S. Stapnes, E. A. Starchenko, G. H. Stark, J. Stark, S. H Stark, P. Staroba, P. Starovoitov, S. Stärz, R. Staszewski, M. Stegler, P. Steinberg, B. Stelzer, H. J. Stelzer, O. Stelzer-Chilton, H. Stenzel, T. J. Stevenson, G. A. Stewart, M. C. Stockton, G. Stoicea, P. Stolte, S. Stonjek, A. Straessner, J. Strandberg, S. Strandberg, M. Strauss, P. Strizenec, R. Ströhmer, D. M. Strom, R. Stroynowski, A. Strubig, S. A. Stucci, B. Stugu, J. Stupak, N. A. Styles, D. Su, J. Su, S. Suchek, Y. Sugaya, M. Suk, V. V. Sulin, D. M. S. Sultan, S. Sultansoy, T. Sumida, S. Sun, X. Sun, K. Suruliz, C. J. E. Suster, M. R. Sutton, S. Suzuki, M. Svatos, M. Swiatlowski, S. P. Swift, A. Sydorenko, I. Sykora, T. Sykora, D. Ta, K. Tackmann, J. Taenzer, A. Taffard, R. Tafirout, E. Tahirovic, N. Taiblum, H. Takai, R. Takashima, E. H. Takasugi, K. Takeda, T. Takeshita, Y. Takubo, M. Talby, A. A. Talyshev, J. Tanaka, M. Tanaka, R. Tanaka, R. Tanioka, B. B. Tannenwald, S. Tapia Araya, S. Tapprogge, A. Tarek Abouelfadl Mohamed, S. Tarem, G. Tarna, G. F. Tartarelli, P. Tas, M. Tasevsky, T. Tashiro, E. Tassi, A. Tavares Delgado, Y. Tayalati, A. C. Taylor, A. J. Taylor, G. N. Taylor, P. T. E. Taylor, W. Taylor, A. S. Tee, P. Teixeira-Dias, D. Temple, H. Ten Kate, P. K. Teng, J. J. Teoh, F. Tepel, S. Terada, K. Terashi, J. Terron, S. Terzo, M. Testa, R. J. Teuscher, S. J. Thais, T. Theveneaux-Pelzer, F. Thiele, J. P. Thomas, A. S. Thompson, P. D. Thompson, L. A. Thomsen, E. Thomson, Y. Tian, R. E. Ticse Torres, V. O. Tikhomirov, Yu. A. Tikhonov, S. Timoshenko, P. Tipton, S. Tisserant, K. Todome, S. Todorova-Nova, S. Todt, J. Tojo, S. Tokár, K. Tokushuku, E. Tolley, M. Tomoto, L. Tompkins, K. Toms, B. Tong, P. Tornambe, E. Torrence, H. Torres, E. Torró Pastor, C. Tosciri, J. Toth, F. Touchard, D. R. Tovey, C. J. Treado, T. Trefzger, F. Tresoldi, A. Tricoli, I. M. Trigger, S. Trincaz-Duvoid, M. F. Tripiana, W. Trischuk, B. Trocmé, A. Trofymov, C. Troncon, M. Trovatelli, F. Trovato, L. Truong, M. Trzebinski, A. Trzupek, F. Tsai, K. W. Tsang, J. C.-L. Tseng, P. V. Tsiareshka, N. Tsirintanis, S. Tsiskaridze, V. Tsiskaridze, E. G. Tskhadadze, I. I. Tsukerman, V. Tsulaia, S. Tsuno, D. Tsybychev, Y. Tu, A. Tudorache, V. Tudorache, T. T. Tulbure, A. N. Tuna, S. Turchikhin, D. Turgeman, I. Turk Cakir, R. Turra, P. M. Tuts, E. Tzovara, G. Ucchielli, I. Ueda, M. Ughetto, F. Ukegawa, G. Unal, A. Undrus, G. Unel, F. C. Ungaro, Y. Unno, K. Uno, J. Urban, P. Urquijo, P. Urrejola, G. Usai, J. Usui, L. Vacavant, V. Vacek, B. Vachon, K. O. H. Vadla, A. Vaidya, C. Valderanis, E. Valdes Santurio, M. Valente, S. Valentinetti, A. Valero, L. Valéry, R. A. Vallance, A. Vallier, J. A. Valls Ferrer, T. R. Van Daalen, W. Van Den Wollenberg, H. Van der Graaf, P. Van Gemmeren, J. Van Nieuwkoop, I. Van Vulpen, M. C. van Woerden, M. Vanadia, W. Vandelli, A. Vaniachine, P. Vankov, R. Vari, E. W. Varnes, C. Varni, T. Varol, D. Varouchas, A. Vartapetian, K. E. Varvell, G. A. Vasquez, J. G. Vasquez, F. Vazeille, D. Vazquez Furelos, T. Vazquez Schroeder, J. Veatch, V. Vecchio, L. M. Veloce, F. Veloso, S. Veneziano, A. Ventura, M. Venturi, N. Venturi, V. Vercesi, M. Verducci, W. Verkerke, A. T. Vermeulen, J. C. Vermeulen, M. C. Vetterli, N. Viaux Maira, O. Viazlo, I. Vichou, T. Vickey, O. E. Vickey Boeriu, G. H. A. Viehhauser, S. Viel, L. Vigani, M. Villa, M. Villaplana Perez, E. Vilucchi, M. G. Vincter, V. B. Vinogradov, A. Vishwakarma, C. Vittori, I. Vivarelli, S. Vlachos, M. Vogel, P. Vokac, G. Volpi, S. E. Von Buddenbrock, E. Von Toerne, V. Vorobel, K. Vorobev, M. Vos, J. H. Vossebeld, N. Vranjes, M. Vranjes Milosavljevic, V. Vrba, M. Vreeswijk, T. Šfiligoj, R. Vuillermet, I. Vukotic, T. Ženiš, L. Živković, P. Wagner, W. Wagner, J. Wagner-Kuhr, H. Wahlberg, S. Wahrmund, K. Wakamiya, J. Walder, R. Walker, W. Walkowiak, V. Wallangen, A. M. Wang, C. Wang, F. Wang, H. Wang, H. Wang, J. Wang, J. Wang, P. Wang, Q. Wang, R.-J. Wang, R. Wang, R. Wang, S. M. Wang, T. Wang, W. Wang, W. X. Wang, Y. Wang, Z. Wang, C. Wanotayaroj, A. Warburton, C. P. Ward, D. R. Wardrope, A. Washbrook, P. M. Watkins, A. T. Watson, M. F. Watson, G. Watts, S. Watts, B. M. Waugh, A. F. Webb, S. Webb, C. Weber, M. S. Weber, S. A. Weber, S. M. Weber, J. S. Webster, A. R. Weidberg, B. Weinert, J. Weingarten, M. Weirich, C. Weiser, P. S. Wells, T. Wenaus, T. Wengler, S. Wenig, N. Wermes, M. D. Werner, P. Werner, M. Wessels, T. D. Weston, K. Whalen, N. L. Whallon, A. M. Wharton, A. S. White, A. White, M. J. White, R. White, D. Whiteson, B. W. Whitmore, F. J. Wickens, W. Wiedenmann, M. Wielers, C. Wiglesworth, L. A. M. Wiik-Fuchs, A. Wildauer, F. Wilk, H. G. Wilkens, H. H. Williams, S. Williams, C. Willis, S. Willocq, J. A. Wilson, I. Wingerter-Seez, E. Winkels, F. Winklmeier, O. J. Winston, B. T. Winter, M. Wittgen, M. Wobisch, A. Wolf, T. M. H. Wolf, R. Wolff, M. W. Wolter, H. Wolters, V. W. S. Wong, N. L. Woods, S. D. Worm, B. K. Wosiek, K. W. Woźniak, K. Wraight, M. Wu, S. L. Wu, X. Wu, Y. Wu, T. R. Wyatt, B. M. Wynne, S. Xella, Z. Xi, L. Xia, D. Xu, H. Xu, L. Xu, T. Xu, W. Xu, B. Yabsley, S. Yacoob, K. Yajima, D. P. Yallup, D. Yamaguchi, Y. Yamaguchi, A. Yamamoto, T. Yamanaka, F. Yamane, M. Yamatani, T. Yamazaki, Y. Yamazaki, Z. Yan, H. J. Yang, H. T. Yang, S. Yang, Y. Yang, Y. Yang, Z. Yang, W-M. Yao, Y. C. Yap, Y. Yasu, E. Yatsenko, K. H. Yau Wong, J. Ye, S. Ye, I. Yeletskikh, E. Yigitbasi, E. Yildirim, K. Yorita, K. Yoshihara, C. J. S. Young, C. Young, J. Yu, J. Yu, X. Yue, S. P. Y. Yuen, I. Yusuff, B. Zabinski, G. Zacharis, R. Zaidan, A. M. Zaitsev, N. Zakharchuk, J. Zalieckas, S. Zambito, D. Zanzi, C. Zeitnitz, G. Zemaityte, J. C. Zeng, Q. Zeng, O. Zenin, D. Zerwas, M. Zgubič, D. F. Zhang, D. Zhang, F. Zhang, G. Zhang, H. Zhang, J. Zhang, L. Zhang, L. Zhang, M. Zhang, P. Zhang, R. Zhang, R. Zhang, X. Zhang, Y. Zhang, Z. Zhang, X. Zhao, Y. Zhao, Z. Zhao, A. Zhemchugov, B. Zhou, C. Zhou, L. Zhou, M. S. Zhou, M. Zhou, N. Zhou, Y. Zhou, C. G. Zhu, H. L. Zhu, H. Zhu, J. Zhu, Y. Zhu, X. Zhuang, K. Zhukov, V. Zhulanov, A. Zibell, D. Zieminska, N. I. Zimine, S. Zimmermann, Z. Zinonos, M. Zinser, M. Ziolkowski, G. Zobernig, A. Zoccoli, K. Zoch, T. G. Zorbas, R. Zou, M. Zur Nedden, L. Zwalinski, ATLAS Collaboration

**Affiliations:** 1 Department of Physics, University of Adelaide, Adelaide, Australia; 2 Physics Department, SUNY Albany, Albany, NY USA; 3 Department of Physics, University of Alberta, Edmonton, AB Canada; 4 Department of Physics, Ankara University, Ankara, Turkey; 5 Istanbul Aydin University, Istanbul, Turkey; 6 Division of Physics, TOBB University of Economics and Technology, Ankara, Turkey; 7 LAPP, Université Grenoble Alpes, Université Savoie Mont Blanc, CNRS/IN2P3, Annecy, France; 8 High Energy Physics Division, Argonne National Laboratory, Argonne, IL USA; 9 Department of Physics, University of Arizona, Tucson, AZ USA; 10 Department of Physics, University of Texas at Arlington, Arlington, TX USA; 11 Physics Department, National and Kapodistrian University of Athens, Athens, Greece; 12 Physics Department, National Technical University of Athens, Zografou, Greece; 13 Department of Physics, University of Texas at Austin, Austin, TX USA; 14 Bahcesehir University, Faculty of Engineering and Natural Sciences, Istanbul, Turkey; 15 Istanbul Bilgi University, Faculty of Engineering and Natural Sciences, Istanbul, Turkey; 16 Department of Physics, Bogazici University, Istanbul, Turkey; 17 Department of Physics Engineering, Gaziantep University, Gaziantep, Turkey; 18 Institute of Physics, Azerbaijan Academy of Sciences, Baku, Azerbaijan; 19 Institut de Física d’Altes Energies (IFAE), Barcelona Institute of Science and Technology, Barcelona, Spain; 20 Institute of High Energy Physics, Chinese Academy of Sciences, Beijing, China; 21 Physics Department, Tsinghua University, Beijing, China; 22 Department of Physics, Nanjing University, Nanjing, China; 23 University of Chinese Academy of Science (UCAS), Beijing, China; 24 Institute of Physics, University of Belgrade, Belgrade, Serbia; 25 Department for Physics and Technology, University of Bergen, Bergen, Norway; 26 Physics Division, Lawrence Berkeley National Laboratory and University of California, Berkeley, CA USA; 27 Institut für Physik, Humboldt Universität zu Berlin, Berlin, Germany; 28 Albert Einstein Center for Fundamental Physics and Laboratory for High Energy Physics, University of Bern, Bern, Switzerland; 29 School of Physics and Astronomy, University of Birmingham, Birmingham, UK; 30 Centro de Investigaciónes, Universidad Antonio Nariño, Bogota, Colombia; 31 Dipartimento di Fisica e Astronomia, Università di Bologna, Bologna, Italy; 32 INFN Sezione di Bologna, Bologna, Italy; 33 Physikalisches Institut, Universität Bonn, Bonn, Germany; 34 Department of Physics, Boston University, Boston, MA USA; 35 Department of Physics, Brandeis University, Waltham, MA USA; 36 Transilvania University of Brasov, Brasov, Romania; 37 Horia Hulubei National Institute of Physics and Nuclear Engineering, Bucharest, Romania; 38 Department of Physics, Alexandru Ioan Cuza University of Iasi, Iasi, Romania; 39 National Institute for Research and Development of Isotopic and Molecular Technologies, Physics Department, Cluj-Napoca, Romania; 40 University Politehnica Bucharest, Bucharest, Romania; 41 West University in Timisoara, Timisoara, Romania; 42 Faculty of Mathematics, Physics and Informatics, Comenius University, Bratislava, Slovakia; 43 Department of Subnuclear Physics, Institute of Experimental Physics of the Slovak Academy of Sciences, Kosice, Slovak Republic; 44 Physics Department, Brookhaven National Laboratory, Upton, NY USA; 45 Departamento de Física, Universidad de Buenos Aires, Buenos Aires, Argentina; 46 Cavendish Laboratory, University of Cambridge, Cambridge, UK; 47 Department of Physics, University of Cape Town, Cape Town, South Africa; 48 Department of Mechanical Engineering Science, University of Johannesburg, Johannesburg, South Africa; 49 School of Physics, University of the Witwatersrand, Johannesburg, South Africa; 50 Department of Physics, Carleton University, Ottawa, ON Canada; 51 Faculté des Sciences Ain Chock, Réseau Universitaire de Physique des Hautes Energies, Université Hassan II, Casablanca, Morocco; 52 Centre National de l’Energie des Sciences Techniques Nucleaires (CNESTEN), Rabat, Morocco; 53 Faculté des Sciences Semlalia, Université Cadi Ayyad, LPHEA-Marrakech, Marrakesh, Morocco; 54 Faculté des Sciences, Université Mohamed Premier and LPTPM, Oujda, Morocco; 55 Faculté des sciences, Université Mohammed V, Rabat, Morocco; 56 CERN, Geneva, Switzerland; 57 Enrico Fermi Institute, University of Chicago, Chicago, IL USA; 58 LPC, Université Clermont Auvergne, CNRS/IN2P3, Clermont-Ferrand, France; 59 Nevis Laboratory, Columbia University, Irvington, NY USA; 60 Niels Bohr Institute, University of Copenhagen, Copenhagen, Denmark; 61 Dipartimento di Fisica, Università della Calabria, Rende, Italy; 62 INFN Gruppo Collegato di Cosenza, Laboratori Nazionali di Frascati, Frascati, Italy; 63 Physics Department, Southern Methodist University, Dallas, TX United States of America; 64 Physics Department, University of Texas at Dallas, Richardson, TX United States of America; 65 Department of Physics, Stockholm University, Stockholm, Sweden; 66 Oskar Klein Centre, Stockholm, Sweden; 67 Deutsches Elektronen-Synchrotron DESY, Hamburg and Zeuthen, Zeuthen, Germany; 68 Lehrstuhl für Experimentelle Physik IV, Technische Universität Dortmund, Dortmund, Germany; 69 Institut für Kern- und Teilchenphysik, Technische Universität Dresden, Dresden, Germany; 70 Department of Physics, Duke University, Durham, NC United States of America; 71 SUPA, School of Physics and Astronomy, University of Edinburgh, Edinburgh, UK; 72 INFN e Laboratori Nazionali di Frascati, Frascati, Italy; 73 Physikalisches Institut, Albert-Ludwigs-Universität Freiburg, Freiburg, Germany; 74 II Physikalisches Institut, Georg-August-Universität Göttingen, Göttingen, Germany; 75 Département de Physique Nucléaire et Corpusculaire, Université de Genève, Genève, Switzerland; 76 Dipartimento di Fisica, Università di Genova, Genoa, Italy; 77 INFN Sezione di Genova, Genoa, Italy; 78 II. Physikalisches Institut, Justus-Liebig-Universität Giessen, Giessen, Germany; 79 SUPA, School of Physics and Astronomy, University of Glasgow, Glasgow, UK; 80 LPSC, Université Grenoble Alpes, CNRS/IN2P3, Grenoble INP, Grenoble, France; 81 Laboratory for Particle Physics and Cosmology, Harvard University, Cambridge, MA United States of America; 82 Department of Modern Physics and State Key Laboratory of Particle Detection and Electronics, University of Science and Technology of China, Hefei, China; 83 Institute of Frontier and Interdisciplinary Science and Key Laboratory of Particle Physics and Particle Irradiation (MOE), Shandong University, Qingdao, China; 84 School of Physics and Astronomy, Shanghai Jiao Tong University, KLPPAC-MoE, SKLPPC, Shanghai, China; 85 Tsung-Dao Lee Institute, Shanghai, China; 86 Kirchhoff-Institut für Physik, Ruprecht-Karls-Universität Heidelberg, Heidelberg, Germany; 87 Physikalisches Institut, Ruprecht-Karls-Universität Heidelberg, Heidelberg, Germany; 88 Faculty of Applied Information Science, Hiroshima Institute of Technology, Hiroshima, Japan; 89 Department of Physics, Chinese University of Hong Kong, Shatin, NT Hong Kong; 90 Department of Physics, University of Hong Kong, Hong Kong, Hong Kong; 91 Department of Physics and Institute for Advanced Study, Hong Kong University of Science and Technology, Clear Water Bay, Kowloon, Hong Kong; 92 Department of Physics, National Tsing Hua University, Hsinchu, Taiwan; 93 Department of Physics, Indiana University, Bloomington, IN USA; 94 INFN Gruppo Collegato di Udine, Sezione di Trieste, Udine, Italy; 95 ICTP, Trieste, Italy; 96 Dipartimento di Chimica, Fisica e Ambiente, Università di Udine, Udine, Italy; 97 INFN Sezione di Lecce, Lecce, Italy; 98 Dipartimento di Matematica e Fisica, Università del Salento, Lecce, Italy; 99 INFN Sezione di Milano, Milan, Italy; 100 Dipartimento di Fisica, Università di Milano, Milan, Italy; 101 INFN Sezione di Napoli, Naples, Italy; 102 Dipartimento di Fisica, Università di Napoli, Naples, Italy; 103 INFN Sezione di Pavia, Pavia, Italy; 104 Dipartimento di Fisica, Università di Pavia, Pavia, Italy; 105 INFN Sezione di Pisa, Pisa, Italy; 106 Dipartimento di Fisica E. Fermi, Università di Pisa, Pisa, Italy; 107 INFN Sezione di Roma, Rome, Italy; 108 Dipartimento di Fisica, Sapienza Università di Roma, Rome, Italy; 109 INFN Sezione di Roma Tor Vergata, Rome, Italy; 110 Dipartimento di Fisica, Università di Roma Tor Vergata, Rome, Italy; 111 INFN Sezione di Roma Tre, Rome, Italy; 112 Dipartimento di Matematica e Fisica, Università Roma Tre, Rome, Italy; 113 INFN-TIFPA, Povo, Italy; 114 Università degli Studi di Trento, Trento, Italy; 115 Institut für Astro- und Teilchenphysik, Leopold-Franzens-Universität, Innsbruck, Austria; 116 University of Iowa, Iowa City, IA USA; 117 Department of Physics and Astronomy, Iowa State University, Ames, IA USA; 118 Joint Institute for Nuclear Research, Dubna, Russia; 119 Departamento de Engenharia Elétrica, Universidade Federal de Juiz de Fora (UFJF), Juiz de Fora, Brazil; 120 Universidade Federal do Rio De Janeiro COPPE/EE/IF, Rio de Janeiro, Brazil; 121 Universidade Federal de São João del Rei (UFSJ), São João del Rei, Brazil; 122 Instituto de Física, Universidade de São Paulo, São Paulo, Brazil; 123 KEK, High Energy Accelerator Research Organization, Tsukuba, Japan; 124 Graduate School of Science, Kobe University, Kobe, Japan; 125 Faculty of Physics and Applied Computer Science, AGH University of Science and Technology, Kraków, Poland; 126 Marian Smoluchowski Institute of Physics, Jagiellonian University, Kraków, Poland; 127 Institute of Nuclear Physics Polish Academy of Sciences, Kraków, Poland; 128 Faculty of Science, Kyoto University, Kyoto, Japan; 129 Kyoto University of Education, Kyoto, Japan; 130 Research Center for Advanced Particle Physics and Department of Physics, Kyushu University, Fukuoka, Japan; 131 Instituto de Física La Plata, Universidad Nacional de La Plata and CONICET, La Plata, Argentina; 132 Physics Department, Lancaster University, Lancaster, UK; 133 Oliver Lodge Laboratory, University of Liverpool, Liverpool, UK; 134 Department of Experimental Particle Physics, Jožef Stefan Institute and Department of Physics, University of Ljubljana, Ljubljana, Slovenia; 135 School of Physics and Astronomy, Queen Mary University of London, London, UK; 136 Department of Physics, Royal Holloway University of London, Egham, UK; 137 Department of Physics and Astronomy, University College London, London, UK; 138 Louisiana Tech University, Ruston, LA USA; 139 Fysiska institutionen, Lunds universitet, Lund, Sweden; 140 Centre de Calcul de l’Institut National de Physique Nucléaire et de Physique des Particules (IN2P3), Villeurbanne, France; 141 Departamento de Física Teorica C-15 and CIAFF, Universidad Autónoma de Madrid, Madrid, Spain; 142 Institut für Physik, Universität Mainz, Mainz, Germany; 143 School of Physics and Astronomy, University of Manchester, Manchester, UK; 144 CPPM, Aix-Marseille Université, CNRS/IN2P3, Marseille, France; 145 Department of Physics, University of Massachusetts, Amherst, MA USA; 146 Department of Physics, McGill University, Montreal, QC Canada; 147 School of Physics, University of Melbourne, Victoria, Australia; 148 Department of Physics, University of Michigan, Ann Arbor, MI USA; 149 Department of Physics and Astronomy, Michigan State University, East Lansing, MI USA; 150 B.I. Stepanov Institute of Physics, National Academy of Sciences of Belarus, Minsk, Belarus; 151 Research Institute for Nuclear Problems of Byelorussian State University, Minsk, Belarus; 152 Group of Particle Physics, University of Montreal, Montreal, QC Canada; 153 P.N. Lebedev Physical Institute of the Russian Academy of Sciences, Moscow, Russia; 154 Institute for Theoretical and Experimental Physics (ITEP), Moscow, Russia; 155 National Research Nuclear University MEPhI, Moscow, Russia; 156 D.V. Skobeltsyn Institute of Nuclear Physics, M.V. Lomonosov Moscow State University, Moscow, Russia; 157 Fakultät für Physik, Ludwig-Maximilians-Universität München, Munich, Germany; 158 Max-Planck-Institut für Physik (Werner-Heisenberg-Institut), Munich, Germany; 159 Nagasaki Institute of Applied Science, Nagasaki, Japan; 160 Graduate School of Science and Kobayashi-Maskawa Institute, Nagoya University, Nagoya, Japan; 161 Department of Physics and Astronomy, University of New Mexico, Albuquerque, NM USA; 162 Institute for Mathematics, Astrophysics and Particle Physics, Radboud University Nijmegen/Nikhef, Nijmegen, The Netherlands; 163 Nikhef National Institute for Subatomic Physics, University of Amsterdam, Amsterdam, The Netherlands; 164 Department of Physics, Northern Illinois University, DeKalb, IL USA; 165 Budker Institute of Nuclear Physics, SB RAS, Novosibirsk, Russia; 166 Novosibirsk State University, Novosibirsk, Russia; 167 Department of Physics, New York University, New York, NY USA; 168 Ohio State University, Columbus, OH USA; 169 Faculty of Science, Okayama University, Okayama, Japan; 170 Homer L. Dodge Department of Physics and Astronomy, University of Oklahoma, Norman, OK USA; 171 Department of Physics, Oklahoma State University, Stillwater, OK USA; 172 Palacký University, RCPTM, Joint Laboratory of Optics, Olomouc, Czech Republic; 173 Center for High Energy Physics, University of Oregon, Eugene, OR USA; 174 LAL, Université Paris-Sud, CNRS/IN2P3, Université Paris-Saclay, Orsay, France; 175 Graduate School of Science, Osaka University, Osaka, Japan; 176 Department of Physics, University of Oslo, Oslo, Norway; 177 Department of Physics, Oxford University, Oxford, UK; 178 LPNHE, Sorbonne Université, Paris Diderot Sorbonne Paris Cité, CNRS/IN2P3 Paris, France; 179 Department of Physics, University of Pennsylvania, Philadelphia, PA USA; 180 Konstantinov Nuclear Physics Institute of National Research Centre “Kurchatov Institute”, PNPI, St. Petersburg, Russia; 181 Department of Physics and Astronomy, University of Pittsburgh, Pittsburgh, PA USA; 182 Laboratório de Instrumentação e Física Experimental de Partículas-LIP, Lisbon, Portugal; 183 Departamento de Física, Faculdade de Ciências, Universidade de Lisboa, Lisbon, Portugal; 184 Departamento de Física, Universidade de Coimbra, Coimbra, Portugal; 185 Centro de Física Nuclear da Universidade de Lisboa, Lisboa, Portugal; 186 Departamento de Física, Universidade do Minho, Braga, Portugal; 187 Departamento de Física Teorica y del Cosmos, Universidad de Granada, Granada, Spain; 188 Dep Física and CEFITEC of Faculdade de Ciências e Tecnologia, Universidade Nova de Lisboa, Caparica, Portugal; 189 Institute of Physics, Academy of Sciences of the Czech Republic, Prague, Czech Republic; 190 Czech Technical University in Prague, Prague, Czech Republic; 191 Faculty of Mathematics and Physics, Charles University, Prague, Czech Republic; 192 State Research Center Institute for High Energy Physics, NRC KI, Protvino, Russia; 193 Particle Physics Department, Rutherford Appleton Laboratory, Didcot, UK; 194 IRFU, CEA , Université Paris-Saclay, Gif-sur-Yvette, France; 195 Santa Cruz Institute for Particle Physics, University of California Santa Cruz, Santa Cruz, CA USA; 196 Departamento de Física, Pontificia Universidad Católica de Chile, Santiago, Chile; 197 Departamento de Física, Universidad Técnica Federico Santa María, Valparaíso, Chile; 198 Department of Physics, University of Washington, Seattle, WA USA; 199 Department of Physics and Astronomy, University of Sheffield, Sheffield, UK; 200 Department of Physics, Shinshu University, Nagano, Japan; 201 Department Physik, Universität Siegen, Siegen, Germany; 202 Department of Physics, Simon Fraser University, Burnaby, BC Canada; 203 SLAC National Accelerator Laboratory, Stanford, CA USA; 204 Physics Department, Royal Institute of Technology, Stockholm, Sweden; 205 Departments of Physics and Astronomy, Stony Brook University, Stony Brook, NY USA; 206 Department of Physics and Astronomy, University of Sussex, Brighton, UK; 207 School of Physics, University of Sydney, Sydney, Australia; 208 Institute of Physics, Academia Sinica, Taipei, Taiwan; 209 Academia Sinica Grid Computing, Institute of Physics, Academia Sinica, Taipei, Taiwan; 210 E. Andronikashvili Institute of Physics, Iv. Javakhishvili Tbilisi State University, Tbilisi, Georgia; 211 High Energy Physics Institute, Tbilisi State University, Tbilisi, Georgia; 212 Department of Physics, Technion: Israel Institute of Technology, Haifa, Israel; 213 Raymond and Beverly Sackler School of Physics and Astronomy, Tel Aviv University, Tel Aviv, Israel; 214 Department of Physics, Aristotle University of Thessaloniki, Thessaloniki, Greece; 215 International Center for Elementary Particle Physics and Department of Physics, University of Tokyo, Tokyo, Japan; 216 Graduate School of Science and Technology, Tokyo Metropolitan University, Tokyo, Japan; 217 Department of Physics, Tokyo Institute of Technology, Tokyo, Japan; 218 Tomsk State University, Tomsk, Russia; 219 Department of Physics, University of Toronto, Toronto, ON Canada; 220 TRIUMF, Vancouver, BC Canada; 221 Department of Physics and Astronomy, York University, Toronto, ON Canada; 222 Division of Physics and Tomonaga Center for the History of the Universe, Faculty of Pure and Applied Sciences, University of Tsukuba, Tsukuba, Japan; 223 Department of Physics and Astronomy, Tufts University, Medford, MA USA; 224 Department of Physics and Astronomy, University of California Irvine, Irvine, CA USA; 225 Department of Physics and Astronomy, University of Uppsala, Uppsala, Sweden; 226 Department of Physics, University of Illinois, Urbana, IL USA; 227 Instituto de Física Corpuscular (IFIC), Centro Mixto Universidad de Valencia - CSIC, Valencia, Spain; 228 Department of Physics, University of British Columbia, Vancouver, BC Canada; 229 Department of Physics and Astronomy, University of Victoria, Victoria, BC Canada; 230 Fakultät für Physik und Astronomie, Julius-Maximilians-Universität Würzburg, Würzburg, Germany; 231 Department of Physics, University of Warwick, Coventry, UK; 232 Waseda University, Tokyo, Japan; 233 Department of Particle Physics, Weizmann Institute of Science, Rehovot, Israel; 234 Department of Physics, University of Wisconsin, Madison, WI USA; 235 Fakultät für Mathematik und Naturwissenschaften, Fachgruppe Physik, Bergische Universität Wuppertal, Wuppertal, Germany; 236 Department of Physics, Yale University, New Haven, CT USA; 237 Yerevan Physics Institute, Yerevan, Armenia; 238 CERN, 1211 Geneva 23, Switzerland

## Abstract

A measurement of $J/\psi$ and $\psi (2\mathrm {S})$ production is presented. It is based on a data sample from Pb+Pb collisions at $\sqrt{s_{\mathrm {NN}}} = 5.02~\mathrm {TeV}$ and *pp* collisions at $\sqrt{s} = 5.02~\mathrm {TeV}$ recorded by the ATLAS detector at the LHC in 2015, corresponding to an integrated luminosity of $0.42~\mathrm {nb}^{-1}$ and $25~\mathrm {pb}^{-1}$ in Pb+Pb and *pp*, respectively. The measurements of per-event yields, nuclear modification factors, and non-prompt fractions are performed in the dimuon decay channel for $9< p_{T}^{\mu \mu } < 40$ GeV in dimuon transverse momentum, and $-2< y_{\mu \mu } < 2$ in rapidity. Strong suppression is found in Pb+Pb collisions for both prompt and non-prompt $J/\psi$, increasing with event centrality. The suppression of prompt $\psi (2\mathrm {S})$ is observed to be stronger than that of $J/\psi$, while the suppression of non-prompt $\psi (2\mathrm {S})$ is equal to that of the non-prompt $J/\psi$ within uncertainties, consistent with the expectation that both arise from *b*-quarks propagating through the medium. Despite prompt and non-prompt $J/\psi$ arising from different mechanisms, the dependence of their nuclear modification factors on centrality is found to be quite similar.

## Introduction

Three decades ago, Matsui and Satz first suggested that charmonia, bound states of *c*- and $\bar{c}$-quarks, could be a sensitive probe to study the hot, dense system created in nucleus–nucleus (A+A) collisions [1]. They postulated that Debye screening of the quark colour charge in a hot plasma would lead to a dissociation of quarkonium bound state in the medium, such as $J/\psi$ or $\psi (2\mathrm {S})$, when the Debye length becomes smaller than the quarkonium binding radius. Therefore, the suppression of the quarkonium production should be significantly larger for $\psi (2\mathrm {S})$ than for $J/\psi$ because the smaller binding energy facilitates the dissociation in the medium. This is referred to as sequential melting [2, 3]. In this picture, the suppression of different quarkonium states could therefore provide information related to the temperature and degree of deconfinement of the medium formed in heavy-ion collisions.

There have been numerous experimental and theoretical investigations since then that have demonstrated that other effects are also present in addition to colour screening in a deconfined plasma [4–6]. First, it has been shown that over a wide range of interaction energies there is already a modification in the production of $J/\psi$ mesons in systems where a large volume of quark–gluon plasma does not appear to form, such as in proton–nucleus collisions [7–9]. Second, it has been shown by the ALICE Collaboration that not only a suppression of quarkonium is observed in ion–ion collisions as reported by several collaborations [10–14], but also an enhancement may play a role leading to an increase in the observed yields of $J/\psi$ at low transverse momentum, $p_{\text {T}}$, relative to higher transverse momenta [15, 16]. This observation has led to the interpretation that recombination of charm quarks and anti-quarks from the medium can play a role by providing an additional mechanism of quarkonium formation [17–19].

Finally, similarities between the suppression of $J/\psi$ and the suppression of charged hadrons and *D*-mesons suggest that high-$p_{\text {T}}$ $J/\psi$s may also be sensitive to parton energy loss in the medium [20, 21]. At LHC energies, $J/\psi$ originates not only from the immediate formation of the composite $c \bar{c}$ bound state (prompt $J/\psi$), but also from the decay of *b*-hadrons, which result in a decay vertex separated from the collision vertex by up to a few millimetres (non-prompt $J/\psi$). When a secondary vertex can be identified, using for instance the precise tracking system of the ATLAS experiment [22], it offers the intriguing possibility of using $J/\psi$ production to study the propagation of *b*-quarks in the hot dense medium. Suppression of the production of *b*-hadrons in the medium, in the most naive picture, is caused by a completely different phenomenon from the suppression of $c \bar{c}$ bound states. While $c\bar{c}$ bound state formation may be inhibited by colour screening from a hot and deconfined medium, the suppression of high-$p_{\text {T}}$ *b*-quark production is commonly attributed to energy loss of propagating *b*-quarks by collisional or radiative processes or both [23], not necessarily suppressing the total cross section but more likely shifting the yield to a lower $p_{\text {T}}$. Quantum interference between the amplitudes for *b*-hadron formation inside and outside of the nuclear medium may also play a role [24].

The modification of prompt $J/\psi$ production is not expected to be similar to the modification of non-prompt $J/\psi$ production, since quite different mechanisms can contribute to those two classes of final states [6]. Simultaneous measurements of prompt and non-prompt charmonia are therefore essential for understanding the physics mechanisms of charmonium suppression in heavy-ion collisions.

This paper reports measurements of prompt and non-prompt per-event yields, non-prompt fraction and nuclear modification factors, $R_{\mathrm {AA}}$, of the $J/\psi$ and $\psi (2\mathrm {S})$. The results are reported for Pb+Pb collisions at $\sqrt{s_{\mathrm {NN}}}$ = 5.02 TeV in the dimuon decay channel and are presented for a 0-80% centrality range, $9< p_{\text {T}} ^{\mu \mu } < 40\ \text {GeV}$ in dimuon transverse momentum, and $-2< y_{\mu \mu } < 2$ in rapidity.

For the quantification of quarkonium suppression in Pb+Pb collisions with respect to *pp* collisions, the cross-section for quarkonium production in $pp$ collisions needs to be measured. This was done in previous ATLAS publication [25].

Section 2 describes the ATLAS detector, Sect. 3 discusses the selection procedure applied to the data, the data analysis is presented in Sect. 4 and systematic uncertainties in Sect. 5. Results and a summary of the paper are presented in Sects. 6 and 7.

## ATLAS detector

The ATLAS detector [22] at the LHC covers nearly the entire solid angle around the collision point.^1^ It consists of an inner tracking detector surrounded by a thin superconducting solenoid, electromagnetic and hadronic calorimeters, and a muon spectrometer incorporating three large superconducting air-core toroid magnets with eight coils each.

The inner-detector system is immersed in a 2 T axial magnetic field and provides charged-particle tracking in the pseudorapidity range $|\eta | < 2.5$. A high-granularity silicon pixel detector covers the vertex region and typically provides three measurements per track, the first hit being normally in the innermost layer. Since 2015 the detector has been augmented by the insertable B-layer [26], an additional pixel layer close to the interaction point which provides high-resolution hits at small radius to improve the tracking and vertex reconstruction performance, significantly contributing to the reconstruction of displaced vertices. It is followed by a silicon microstrip tracker which comprises eight cylindrical layers of single-sided silicon strip detectors in the barrel region, and nine disks in the endcap region. These silicon detectors are complemented by a transition radiation tracker (TRT), which enables radially extended track reconstruction up to $|\eta | = 2.0$.

The calorimeter system covers the pseudorapidity range $|\eta | < 4.9$. Within the region $|\eta |< 3.2$, electromagnetic calorimetry is provided by barrel and endcap high-granularity lead/liquid-argon (LAr) calorimeters, with an additional thin LAr presampler covering $|\eta | < 1.8$, to correct for energy loss in material upstream of the calorimeters. Hadronic calorimetry is provided by a steel/scintillator-tile calorimeter, segmented into three barrel structures within $|\eta | < 1.7$, and two copper/LAr hadronic endcap calorimeters situated at $1.5< |\eta | < 3.2$. The solid angle coverage is completed with forward copper/LAr and tungsten/LAr calorimeter modules (FCal) situated at $3.1< |\eta | < 4.9$, optimized for electromagnetic and hadronic measurements respectively.

The muon spectrometer comprises separate trigger and high-precision tracking chambers measuring the deflection of muons in a magnetic field generated by the superconducting air-core toroids. The precision chamber system covers the region $|\eta | < 2.7$ with three layers of monitored drift tubes, complemented by cathode strip chambers in the forward region, where the background is the highest. The muon trigger system covers the range of $|\eta | < 2.4$ with resistive plate chambers in the barrel, and thin gap chambers in the endcap regions.

In addition to the muon trigger, two triggers are used in Pb+Pb collisions to select minimum-bias events for the centrality characterization. These are based on the presence of a minimum amount of transverse energy in all sections of the calorimeter system ($|\eta | < 3.2$) or, for events which do not meet this condition, on the presence of substantial energy deposits in both zero-degree calorimeters (ZDCs), with a threshold set just below the one-neutron peak, which are primarily sensitive to spectator neutrons in the region $|\eta | > 8.3$. Those two triggers were found to be fully efficient in the centrality range studied in this analysis.

A two-level trigger system is used to select events of interest [27]. The first-level (L1) trigger is implemented in hardware and uses a subset of detector information to reduce the event rate to a design value of at most 100 kHz. This is followed by a software-based high-level trigger (HLT), which reduces the event rate to a maximum value of 1 kHz.

## Event and data selection

The analysis presented in this paper uses data from Pb+Pb collisions at a nucleon–nucleon centre-of-mass energy of $\sqrt{s_{_\text {NN}}}$ = 5.02 TeV and $pp$ collisions at a centre-of-mass energy of $\sqrt{s}$ = 5.02 TeV recorded by the ATLAS experiment in 2015. The integrated luminosity of previously analysed $pp$ sample is $25~\text{ pb }^{-1}$. The integrated luminosity of Pb+Pb sample is $0.42~\mathrm {nb^{-1}}$.

Events were collected using a trigger requiring that the event contains at least two reconstructed muons. In the previously analysed $pp$ sample both muons must generate a L1 muon trigger and be confirmed by the HLT while in the Pb+Pb sample only one muon is required to be seen by the L1 muon trigger and confirmed by the HLT; the second muon is only required to pass the HLT. At both levels the muon must satisfy the requirement of $p_\mathrm {T} > 4$ GeV, as reconstructed by the trigger system.

Monte Carlo (MC) simulations are used for performance studies, where the response of the ATLAS detector was simulated using Geant 4 [28, 29]. Prompt ($$pp \rightarrow J/\psi \rightarrow \mu \mu $$) and non-prompt ($$pp \rightarrow b\bar{b} \rightarrow J/\psi \rightarrow \mu \mu $$) samples of $$J/\psi $$ were produced with the event generator Pythia 8.212 [30] and corrected for electromagnetic radiation with Photos [31]. The A14 set of tuned parameters [32] is used together with the CTEQ6L1 parton distribution function set [33]. These samples were used to study the trigger and reconstruction performance of the $$pp $$ collisions. In order to simulate $$J/\psi $$ production in the high multiplicity environment of Pb+Pb collisions, the generated events were overlaid with a sample of minimum-bias events produced with HIJING [34].

Muon candidates are required to pass the “tight” muon working point selection [35] without any TRT requirements, have $p_{\text {T}} >4$ GeV, and $|\eta |<2.4$ in addition to being the reconstructed muon associated, in $\Delta R<0.01$, with the trigger decision. To be selected, a muon pair must be consistent with originating from a common vertex, have opposite charge, and an invariant mass in the range $2.6<m_{\mu \mu }<4.2$ GeV. The dimuon candidate is further required to have $p_\text {T}^{\mu \mu } > 9$ GeV to ensure that the pair candidates are reconstructed in a fiducial region where systematic uncertainties in the final results do not vary significantly relative to the acceptance and efficiency corrections.

The centrality of Pb+Pb collisions is characterized by the sum of the transverse energy, $\sum E_\text {T}^\mathrm {FCal}$, evaluated at the electromagnetic scale (that is before hadronic calibration) in the FCal. It describes the degree of geometric overlap of two colliding nuclei in the plane perpendicular to the beam with large overlap in central collisions and small overlap in peripheral collisions. Centrality intervals are defined in successive percentiles of the $\sum E_\text {T}^\mathrm {FCal}$ distribution ordered from the most central (highest $\sum E_\text {T}^\mathrm {FCal}$ ) to the most peripheral collisions. A Glauber model analysis of the $\sum E_\text {T}^\mathrm {FCal}$ distribution was used to evaluate the mean nuclear thickness function, $\langle T_{\text {AA}} \rangle$, and the number of nucleons participating in the collision, $\langle N_{\text {part}} \rangle$, in each centrality interval [36–38]. The centrality intervals used in this measurement are indicated in Table 1 along with their respective calculations of $\langle T_{\text {AA}} \rangle$ and $\langle N_{\text {part}} \rangle$.

**Table 1:** The $\langle T_{\text {AA}} \rangle$, $\langle N_{\text {part}} \rangle$ values and their uncertainties in each centrality bin. These are the results from the Glauber modelling of the summed transverse energy in the forward calorimeters, $\sum E_\text {T}^\mathrm {FCal}$

Centrality (%)	$\langle T_{\text {AA}} \rangle$ ($\mathrm {mb}^{-1}$)	$\langle N_{\text {part}} \rangle$
0–5	26.23 ± 0.22	384.4 ± 1.9
5–10	20.47 ± 0.19	333.1 ± 2.7
0–10	23.35 ± 0.20	358.8 ± 2.3
10–20	14.33 ± 0.17	264.0 ± 2.8
20–30	8.63 ± 0.17	189.1 ± 2.7
30–40	4.94 ± 0.15	131.4 ± 2.6
40–50	2.63 ± 0.11	87.0 ± 2.3
50–60	1.27 ± 0.07	53.9 ± 1.9
60–80	0.39 ± 0.03	22.9 ± 1.2
20–50	5.40 ± 0.14	135.8 ± 2.5
0–80	6.99 ± 0.10	141.3 ± 2.0

The number of minimum-bias events, $N_{\text {evt}}$, times the centrality fraction, is used to normalize the yield in respective centrality class. Minimum-bias events are selected by requiring that they pass at least one of the two minimum-bias triggers. The analysed dataset corresponds, after correction for the trigger prescale factor, to $2.99 \times 10^{9}$ Pb+Pb minimum bias events.

## Data analysis

The pseudo-proper decay time, $\tau$, is used to distinguish between prompt and non-prompt charmonium production. It is defined as,

$$\begin{aligned} \tau = \frac{L_{xy}m_{\mu \mu }}{p_{\text {T}} ^{\mu \mu }}, \end{aligned}$$

where $L_{xy}$ is the distance between the position of the reconstructed dimuon vertex and the primary vertex projected onto the transverse plane. A weight, $w_{\mathrm {total}}$, is defined for each selected dimuon candidate using the relation:

$$\begin{aligned} w^{-1}_{\mathrm {total}} = A\times \epsilon _{\mathrm {reco}}\times \epsilon _{\mathrm {trig}}, \end{aligned}$$

where *A* is the acceptance, $\epsilon _{\mathrm {reco}}$ is the reconstruction efficiency, and $\epsilon _{\mathrm {trig}}$ is the trigger efficiency.

A two-dimensional unbinned maximum-likelihood fit to the invariant mass and pseudo-proper time distributions of weighted events is used to determine the yields of the prompt and non-prompt charmonium components as well as the contribution from background. A total of 31 572 events before applying the weights are used in the fit.

The differential cross sections for the production of prompt (p) and non-prompt (np) $J/\psi$ and $\psi (2\mathrm {S})$ in $pp$ collisions were calculated in a previously published study [25] and are defined as:

$$\begin{aligned} \frac{\text {d}^2\sigma ^\text {p(np)}}{\text {d}p_{\text {T}} \text {d}y}\times B(\psi (\textit{n}\mathrm {S})\rightarrow \mu \mu ) = \frac{N_{\psi (\textit{n}\mathrm {S})}^\mathrm {p(np),~corr}}{\Delta p_{\text {T}} \times \Delta y\times \int {\mathcal {L}dt}}, \end{aligned}$$

where $B(\psi (\textit{n}\mathrm {S})\rightarrow \mu \mu )$ is the branching ratio for charmonium states decaying into two muons [39], $N_{\psi (\textit{n}\mathrm {S})}^\mathrm {p(np),~corr}$ is the prompt and non-prompt charmonium yield corrected for acceptance and detector effects, and $\Delta p_{\text {T}}$ and $\Delta y$ are the widths of the $p_{\text {T}}$ and *y* bins. Following the same approach, the per-event yield of charmonium states measured in A+A collisions is calculated as:

1 $$\begin{aligned} \left. \frac{\text {d}^{2}N^\text {p(np)}}{\text {d}p_{\text {T}} \text {d}y}\right| _\text {cent} \times B(\psi (\textit{n}\mathrm {S})\rightarrow \mu \mu ) = \frac{1}{\Delta p_{\text {T}} \times \Delta y}\times \left. \frac{N_{\psi (\textit{n}\mathrm {S})}^\mathrm {p(np),~corr}}{N_\text {evt}}\right| _\text {cent}, \end{aligned}$$

where $N_\text {evt}$ is the number of minimum-bias events and “cent” refers to a specific centrality class.

### Acceptance and efficiency corrections

The kinematic acceptance $A(p_{\text {T}},y)$ for a $\psi (\textit{n}\mathrm {S})$ with transverse momentum $p_{\text {T}}$ and rapidity *y* decaying into $\mu \mu$ was obtained from a MC simulation and is defined as the probability that both muons fall within the fiducial volume $p_\text {T}(\mu ^{\pm })>4$ GeV and $|\eta (\mu ^{\pm })|<2.4$. Acceptance generally depends on the $\psi (\textit{n}\mathrm {S})$ polarization. In this study, we assume that the $\psi (\textit{n}\mathrm {S})$ are unpolarized following Refs. [40–42]. The effects of variations to this assumption have been considered and are discussed in Sect. 5. In order to apply the acceptance weight to each charmonia candidate, a simple linear interpolation is used in the mass range where the $J/\psi$ and $\psi (2\mathrm {S})$ overlap due to the detector resolution. The upper mass boundary for the $J/\psi$ candidates is chosen to be 3.5 GeV and the lower mass boundary for the $\psi (2\mathrm {S})$ candidates to be 3.2 GeV, resulting in a superposition range of 0.3 GeV. Within the interpolation range of $m_{\mu \mu }$ = 3.2–3.5 GeV, the following function was applied for the acceptance correction:

2 $$\begin{aligned} A =A(J/\psi ) \times \frac{3.5 - m_{\mu \mu }}{0.3} + A(\psi (2\mathrm {S})) \times \frac{m_{\mu \mu } - 3.2}{0.3}. \end{aligned}$$

The difference between the $J/\psi$ and $\psi (2\mathrm {S})$ acceptance varies from 5% at low $p_{\text {T}}$ to 0.05% at high $p_{\text {T}}$.

Trigger and reconstruction efficiencies were calculated for both data and MC simulation using the tag-and-probe (T&P) method. The method is based on the selection of an almost pure muon sample from $J/\psi \rightarrow \mu \mu$ events collected with an auxiliary single-muon trigger, requiring one muon of the decay (tag) to be identified as the “tight” muon which triggered the read-out of the event and the second muon (probe) to be reconstructed as a system independent of the one being studied, allowing a measurement of the performance with minimal bias. Once the tag and probe sample is defined, the background contamination and the muon efficiency are measured with a simultaneous maximum-likelihood fit of two statistically independent distributions of the invariant mass: events in which the probe is or is not successfully matched to the selected muon [35, 43]. Both efficiencies were evaluated as a function of $p_{\text {T}}$ and $\eta$, in narrow bins, using muons from simulated $J/\psi \rightarrow \mu \mu$ decays in order to build the efficiency map. Muon reconstruction efficiency increases from low to high $p_{\text {T}}$ and decreases from central to forward rapidities. It varies between 60% and 90%, becoming almost constant for $p_{\text {T}} >6$ GeV. The dimuon trigger efficiency is studied and factorized in terms of single-muon trigger efficiencies which increase from low to high $p_{\text {T}}$ and from central to forward rapidities. Dimuon trigger efficiency increases from 50% to 85% between the lowest and highest dimuon $p_{\text {T}}$.

**Fig. 1:**
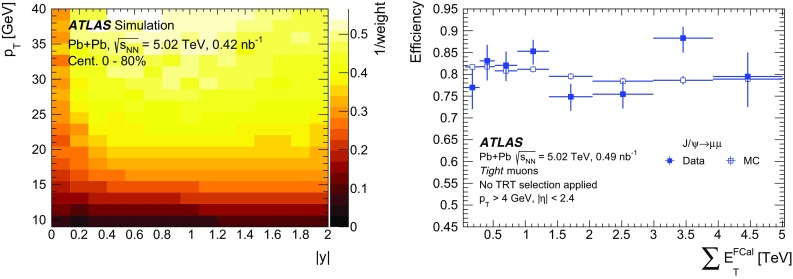
(Left) Inverse total weight binned in the dimuon transverse momentum and rapidity for integrated centrality as estimated in MC simulation and corrected for differences between efficiencies in MC and experimental data. Decreases in efficiency at very central rapidity correspond to the $|\eta |<0.1$ region not covered by the muon detectors. The weight is dominated by the acceptance correction. (Right) Muon reconstruction efficiency as a function of the summed transverse energy in the forward calorimeters, $\sum E_\text {T}^\mathrm {FCal}$

In order to account for the difference between efficiencies in simulation and experimental data, the data-to-MC ratio, $\epsilon ^\mathrm {data}_{\mathrm {reco}}/\epsilon ^{\mathrm {MC}}_{\mathrm {reco}}$, was parameterized as a function of $p_\text {T}$ and centrality and applied as a multiplicative scale factor to the efficiency correction separately for the barrel and endcap regions of the muon spectrometer. This scale factor varies between 1.01 and 1.05. The inverse total weight, $w^{-1}_{\mathrm {total}}$, after applying the scale factor, is shown in the left panel of Fig. 1, averaged in bins of the dimuon transverse momentum and rapidity. The right panel of Fig. 1 shows the centrality dependence of the muon reconstruction efficiency.

### Fit model

The corrected prompt and non-prompt $\psi (\textit{n}\mathrm {S})$ yields are extracted from two-dimensional weighted unbinned maximum-likelihood fits performed on invariant mass and pseudo-proper decay time distributions. A fit is made for each $p_{\text {T}}$, *y*, and centrality interval measured in this analysis. The probability distribution function (PDF) for the fit [44] is defined as a normalized sum of seven terms listed in Table 2, where each term is factorized into mass-dependent and decay-time-dependent functions; these functions are described below. The PDF can be written in a compact form as:

$$\begin{aligned} \text {PDF}(m,\tau ) = \sum ^7_{i=1}\kappa _i f_i(m) \cdot h_i(\tau ) \otimes g(\tau ), \end{aligned}$$

where $\kappa _i$ is the normalization factor of each component, $f_i(m)$ and $h_i(\tau )$ are distribution functions for the mass *m* and the pseudo-proper time $\tau$ respectively; $g(\tau )$ is the resolution function described with a sum of two Gaussian distribution; and the “$\otimes$” symbol denotes a convolution. The distribution functions $f_i$ and $h_i$ are defined by a Crystal Ball ($C\!B$) function [45], Gaussian (*G*), Dirac delta ($\delta$) and exponential (*E*) distributions; individual components are shown in Table 2. The fit is performed using the RooFit framework [46]. In order to stabilize the fit model, and reduce the correlation between parameters, a number of component terms listed in Table 2 share common parameters, are scaled to each other by a multiplicative scaling parameter, or are fixed to the value observed in MC simulation.

**Table 2:** Probability distribution functions for individual components in the default fit model used to extract the prompt (p) and non-prompt (np) contribution for $J/\psi$ and $\psi (2\mathrm {S})$ signal and background (Bkg). Symbols denote functions as follows: “$C\!B$” – Crystal Ball, “*G*” – Gaussian, “*E*” – exponential, and “$\delta$” – Dirac delta function

*i*	Type	Source	$f_i(m)$	$h_i(\tau )$
1	$J/\psi$	p	$\omega \ C\!B_1(m)+(1-\omega )G_1(m)$	$\delta (\tau )$
2	$J/\psi$	np	$\omega \ C\!B_1(m)+(1-\omega )G_1(m)$	$E_1(\tau )$
3	$\psi (2\mathrm {S})$	p	$\omega \ C\!B_2(m)+(1-\omega )G_2(m)$	$\delta (\tau )$
4	$\psi (2\mathrm {S})$	np	$\omega \ C\!B_2(m)+(1-\omega )G_2(m)$	$E_2(\tau )$
5	Bkg	p	$E_3(m)$	$\delta (\tau )$
6	Bkg	np	$E_4(m)$	$E_5(\tau )$
7	Bkg	np	$E_6(m)$	$E_7(|\tau |)$

The signal mass shapes of the $J/\psi$ and $\psi (2\mathrm {S})$ are each described by the sum of a $C\!B$ function, which covers the $J/\psi$ invariant mass distribution’s low-side tail due to final-state radiation, and a single Gaussian function which share a common peak position treated as a free parameter. The width term in the $C\!B$ function is equal to the Gaussian standard deviation times a free scaling term that is common to the $J/\psi$ and $\psi (2\mathrm {S})$. The $C\!B$ low-mass tail and height parameters are fixed to the MC value. Variations of these two parameters are considered a part of the fit model’s systematic uncertainties. The mean of the $\psi (2\mathrm {S})$ mass profile is set to be the mean of the $J/\psi$ mass profile multiplied by the ratio of their known masses, $m_{\psi (2\mathrm {S})}/m_{J/\psi } = 1.190$ [39]. The Gaussian width of the $\psi (2\mathrm {S})$ is also set to be the width of the $J/\psi$ multiplied by the same factor. Variations of this scaling term are considered a part of the fit model systematic uncertainties. The relative fraction of the $C\!B$ and Gaussian functions, $\omega$, is free but common to the $J/\psi$ and $\psi (2\mathrm {S})$.

The non-prompt signal pseudo-proper decay time PDFs are described by a single-sided exponential function (for positive $\tau$ only) convolved with a sum of two Gaussians lifetime resolution function. The sum of two Gaussian resolution function has a fixed mean at $\tau = 0$ and free widths with a fixed relative fraction for the two single Gaussian components. The same resolution function is used to describe the prompt contribution by convolving it with a delta function.

The pseudo-proper decay time PDFs describing the background are represented by the sum of one prompt component and two non-prompt components. The prompt background component is described by a delta function convolved with a sum of two Gaussian function. While one of the non-prompt background contributions is described by a single-sided decay model (for positive $\tau$ only), the other is described by a double-sided decay model accounting for candidates of mis-reconstructed or non-coherent dimuon pairs resulting from Drell–Yan muons and combinatorial background. The same Gaussian resolution functions are used for the background and the signal. For the background parameterizations in the mass distribution, the three components: prompt, single-sided non-prompt, and double-sided non-prompt were modelled with exponentials functions.

**Fig. 2:**
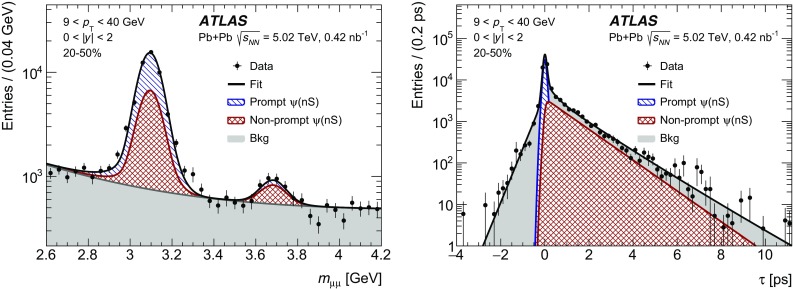
Dimuon invariant mass for events with $2.6< m_{\mu \mu } < 4.2\ \text {GeV}$ (left) and dimuon pseudo-proper lifetime (right). The data, corrected for acceptance times efficiency, are shown for the range $9< p_{\text {T}} < 40\ \text {GeV}$, $|y| < 2.0$, and centrality 20–50% in Pb+Pb collisions. Superimposed on the data are the projections of the fit results

Example fit projections are shown in Fig. 2. The important quantities extracted from the fit are: the number of signal $J/\psi$, the number of signal $\psi (2\mathrm {S})$, the non-prompt fraction of the $J/\psi$ signal, and the non-prompt fraction of the $\psi (2\mathrm {S})$ signal. From these values and the correlation matrix of the fit, all the measured observables and their uncertainties are extracted.

### Observables

The suppression of charmonium states is quantified by the nuclear modification factor, which can be defined for a given centrality class as:

3 $$\begin{aligned} R_{\mathrm {AA}} = \frac{N_{\mathrm {AA}}}{\langle T_{\mathrm {AA}}\rangle \times \ \sigma _{pp}}, \end{aligned}$$

where $$N_\mathrm {AA}$$ is the per-event yield of charmonium states measured in A+A collisions, $$\langle T_\mathrm {AA}\rangle $$ is the mean nuclear thickness function and $$\sigma _{pp}$$ is the cross section for the production of the corresponding charmonium states in $$pp $$ collisions at the same energy [25].

In order to quantify the production of $\psi (\mathrm {2\mathrm {S}})$ relative to $J/\psi$ a ratio of nuclear modification factors, $\rho _\text {PbPb}^{\psi (2\mathrm {S})/J/\psi }$ = $R_\mathrm {AA}^{\psi (\mathrm {2\mathrm {S}})}/R_\mathrm {AA}^{J/\psi }$, can be used. However, in this analysis the numerator and denominator are not calculated directly from Eq. (3), rather, it is advantageous to calculate it in the equivalent form as:

$$\begin{aligned} \rho _\text {PbPb}^{\psi (2\mathrm {S})/J/\psi } = ( N_{\psi (2\mathrm {S})} / N_{J/\psi } )_{\text{ Pb }+\text{ Pb }} /( N_{\psi (2\mathrm {S})} / N_{J/\psi } )_{pp}. \end{aligned}$$

This formulation minimizes the systematic uncertainties due to a substantial cancelling-out of the trigger and reconstruction efficiencies for the two quarkonium systems because they are very similar in mass and they are measured in the identical final-state channel.

Also measured is the non-prompt fraction $f_\mathrm {np}$, which is defined as the ratio of the number of non-prompt charmonia to the number of inclusively produced charmonia,

$$\begin{aligned} f_\mathrm {np}^{\psi (\textit{n}\mathrm {S})} = \frac{N_{\psi (\textit{n}\mathrm {S})}^\mathrm {np,corr}}{N_{\psi (\textit{n}\mathrm {S})}^\mathrm {np,corr} + N_{\psi (\textit{n}\mathrm {S})}^\mathrm {p,corr}}, \end{aligned}$$

where the non-prompt fraction can be determined for the $J/\psi$ and $\psi (2\mathrm {S})$ simultaneously. This observable has the advantage that acceptances and efficiencies are similar for the numerator and denominator, and thus systematic uncertainties are reduced in the ratio.

## Systematic uncertainties

The main sources of systematic uncertainty in this measurement are the assumptions in the fitting procedure, the acceptance and efficiency calculations, and the *pp* luminosity and $\langle T_\mathrm {AA}\rangle$ determination. The acceptance, and hence the corrected yields, depend on the spin-alignment state of the $\psi (\textit{n}\mathrm {S})$. For prompt production, six alternative scenarios have been considered, corresponding to extreme cases of spin alignment, as explained in Ref. [44]. An envelope to the acceptance has been obtained from the maximum deviations from the assumption of unpolarized production. In the non-prompt case a map weighted to the CDF result [47] for $B\rightarrow J/\psi$ spin-alignment is used as a variation. Since the polarization of charmonia in *pp* collisions was measured to be small [40–42], its modification due to the nuclear environment is neglected and the spin-alignment uncertainty is assumed to cancel out in $R_{\text{ AA }}$ and $\rho _\text {PbPb}^{\psi (2\mathrm {S})/J/\psi }$. Changes in the yields due to bin migration effects are at the per-mil level and thus no correction is needed. Table 3 shows the systematic uncertainties affecting the three measured observables. The total systematic uncertainty is calculated by summing the different contributions in quadrature and is derived separately for *pp* and Pb+Pb results. No differences in the uncertainties was observed for prompt and non-prompt production. The yield extraction uncertainties, which are dominated by the uncertainty in the muon reconstruction, increase from central to forward rapidity, and from high to low $p_{\text {T}}$. The double $R_\mathrm {AA}$ ratio, $\rho _\text {PbPb}^{\psi (2\mathrm {S})/J/\psi }$ has a substantially larger fit uncertainty than the other observables; this is because the signal-to-background ratio for the $\psi (2\mathrm {S})$ is much smaller than for the $J/\psi$. For $R_\mathrm {AA}$ and $\rho _\text {PbPb}^{\psi (2\mathrm {S})/J/\psi }$ the correlations between the uncertainty in the *pp* and Pb+Pb samples are taken into account.

**Table 3:** Systematic uncertainties of the $J/\psi$ yield, $R^{J/\psi }_\text {AA}$ and $\rho _\text {PbPb}^{\psi (2\mathrm {S})/J/\psi }$ measured in Pb+Pb collisions. “Uncorr.” refers to point-to-point uncorrelated uncertainties and “Corr.” refers to global uncertainties from various sources

Source	$J/\psi$ yield		$R^{J/\psi }_\text {AA}$		$\rho _\text {PbPb}^{\psi (2\mathrm {S})/J/\psi }$
	Uncorr. (%)	Corr. (%)	Uncorr. (%)	Corr. (%)	Uncorr. (%)
Trigger	2–4	3	5–6	5	$<1$
Reconstruction	4–5	2	6–7	2	$<1$
Fitting	1–2	1	1–2	1	8–9
$T_{\text {AA}}$	–	1–8	–	1–8	–
Luminosity	–	–	–	5.4	–

### Proton–proton luminosity and mean nuclear thickness uncertainties

The integrated luminosity determined for the 2015 $$pp$$ data was calibrated using data from dedicated beam-separation scans, also known as van der Meer scans. Sources of systematic uncertainty similar to those examined in the 2012 $$pp $$ luminosity calibration [48] were studied in order to assess the systematic uncertainties for the 2015 data. The combination of these systematic uncertainties results in a uncertainty in the luminosity during $$pp$$ collisions at $$\sqrt{s} = 5.02\ \text {TeV}$$ of $$\delta \mathcal {L}/\mathcal {L} = \pm 5.4\%$$. The uncertainty in the value of the nuclear overlap function $$\langle T_{\text {AA}} \rangle $$ is estimated by varying the Glauber model parameters [38] and is shown in Table 1. This uncertainty is treated as fully correlated across $$p_{\text {T}}$$ and *y* bins for the same centrality and it is reported separately from other uncertainties. For the case of the $$R_{\text{ AA }}$$ evaluated as a function of $$N_{\text {part}}$$, the $$T_{\text {AA}}$$ uncertainty is added in quadrature with other uncertainties.

### Trigger and reconstruction efficiency uncertainty

Several sources of systematic uncertainty were examined to assess the uncertainties of the muon efficiency determination. The statistical uncertainty of the fitted scale factors is propagated as a systematic uncertainty. The signal and background fit models used to extract the data efficiency in the T&P method are changed to assess systematic uncertainties related to the choice of signal and background PDFs. A Chebychev polynomial is used instead of an exponential function for the background model variation, and a single Gaussian function is used instead of a weighted sum of Gaussian and CB functions for the signal mass resolution model variation.

For the reconstruction efficiency, the difference between the “true” muon efficiency given by the fraction of generator-level muons that are successfully reconstructed and the efficiency determined using the T&P method in MC simulation is also assigned as a correlated systematic uncertainty. The accuracy of dimuon chain factorization was estimated using MC simulation. The difference between the initial number of dimuons in the sample and the number of dimuons after trigger selection and correction was assessed as the systematic uncertainty, having a value of 3%. The centrality-dependent corrections have an uncertainty of $\mathcal {O}$(1%). These uncertainties apply to the cross sections but most cancel out in the ratios of $\psi (2\mathrm {S})$ to $J/\psi$ yields, leaving a residual difference of less than 1%.

### Fit model uncertainty

The uncertainty associated with the particular choice of PDFs was evaluated by varying the PDF of each component, using ten alternative models. In each variation of the fit model, all measured quantities were recalculated and compared to the nominal fit. The root mean square of all variations was then assigned as the fit model’s systematic uncertainty. The signal mass PDF was varied by replacing the $C\!B$ plus Gaussian function with a double Gaussian function, and varying parameters of the $C\!B$ model, which were originally fixed. For the signal decay time PDF, a single exponential function was changed to a sum of two exponential function. The background mass PDFs were varied by replacing exponential functions with second-order Chebyshev polynomials in order to describe the prompt, non-prompt and double-sided background terms. Finally, the decay time resolution was varied by using a single Gaussian function in place of the double Gaussian function.

The stability of the nominal fitting procedure is quantified by comparing the yield of a randomly weighted MC simulation sample of prompt and non-prompt $J/\psi$ with the fit output of the same sample. The comparison shows a 1% difference in the yield extractions and non-prompt fraction. This is assigned as an additional systematic uncertainty in the yields and non-prompt fraction value, which, however, cancels out in the $\psi$(2S) to $J/\psi$ ratio. An extra systematic uncertainty is added to the $\psi$(2S) to $J/\psi$ ratio to account for a $2\%$ bias introduced by the acceptance interpolation (see Eq. (2)). This value comes from comparing the fit results from a sample that is corrected with a standalone acceptance and other that used the interpolation. The difference between both samples was found to be significant only when the signal-to-background ratio was small, which is typical for the $\psi$(2S).

## Results

### Prompt and non-prompt $J/\psi$ per-event yields for Pb+Pb collisions

**Fig. 3:**
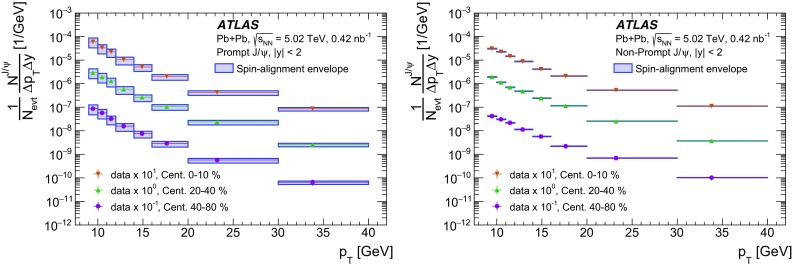
Pb+Pb per-event yields of prompt $J/\psi$ (left) and non-prompt $J/\psi$ (right) as a function of $p_{\text {T}}$ for three different centrality slices in the rapidity range $|y| < 2$. The centroids of the $p_{\text {T}}$ bins are the mean value of the transverse momentum distributions of dimuons in the $J/\psi$ mass region, corrected for acceptance $\times$ efficiency. The vertical error bars are the combined systematic and statistical uncertainties, where the dominant source is the systematic uncertainty with the exception of the latest bin. Overlaid is a band representing the variation of the result in various spin-alignment scenarios

**Fig. 4:**
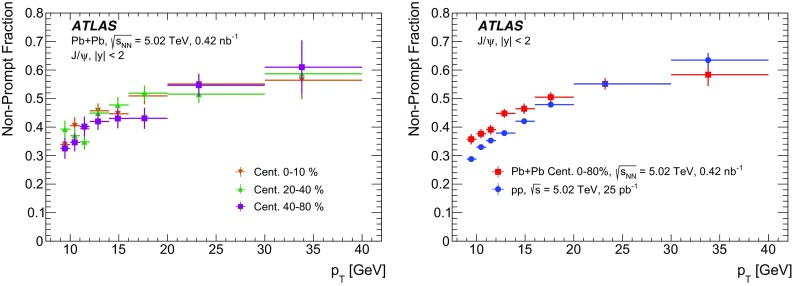
(Left) Non-prompt fraction of $J/\psi$ production in 5.02 TeV Pb+Pb collision data as a function of $p_{\text {T}}$ for three different centrality slices in the rapidity range $|y| < 2$. (Right) Comparison with the ATLAS 5.02 TeV $pp$ collision data [25]. The vertical error bars are the combined systematic and statistical uncertainties, dominated by the statistical uncertainty

The per-event yields are defined as the number of $J/\psi$ produced per bin of $p_{\text {T}}$, *y* and centrality intervals normalized by the width of the $p_{\text {T}}$ and *y* bin and the number of events, $N_\text {evt}$, measured in minimum-bias data for each centrality class, as defined in Eq. (1). The resulting per-event yields and non-prompt fraction for $J/\psi$ production are shown in Figs. 3 and 4 respectively, as a function of transverse momentum, for three centrality slices and rapidity range $|y|<2$. The vertical error bars in the $J/\psi$ per-event yields shown in Fig. 3 are the combined systematic and statistical uncertainties. The non-prompt fraction appears to be essentially centrality-independent and to have a slightly different slope from that found in *pp* collisions [25].

### Nuclear modification factor, $R^{J/\psi }_\mathrm {AA}$

**Fig. 5:**
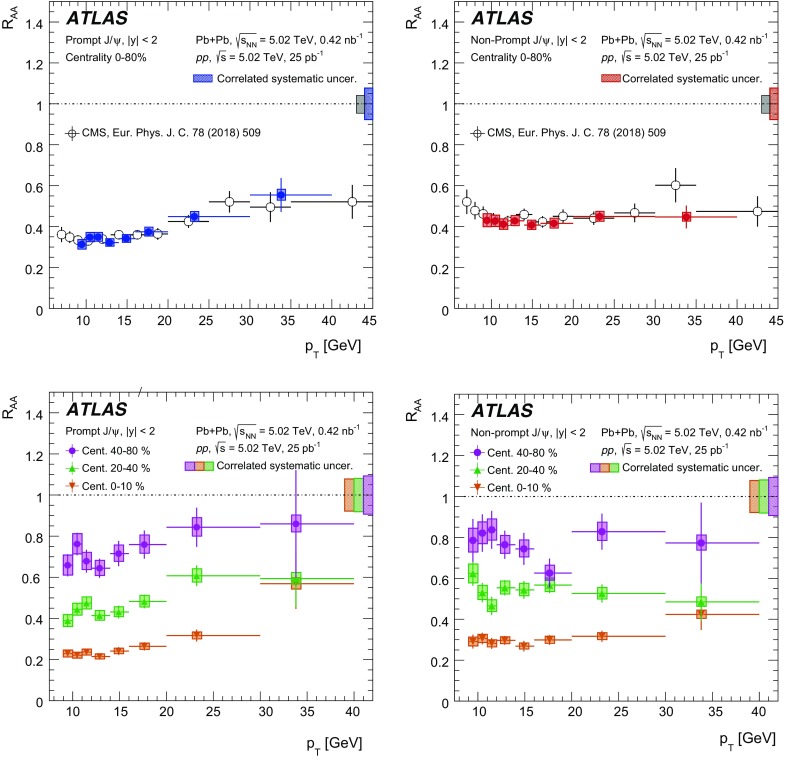
The nuclear modification factor as a function of $p_{\text {T}}$ for the prompt $J/\psi$ (left) and non-prompt $J/\psi$ (right) for $|y|<2$, in 0–80% centrality bin (top) and in 0–10%, 20–40%, and 40–80% centrality bins (bottom). The statistical uncertainty of each point is indicated by a narrow error bar. The error box plotted with each point represents the uncorrelated systematic uncertainty, while the shaded error box at $R_{\mathrm {AA}}$=1 represents correlated scale uncertainties

**Fig. 6:**
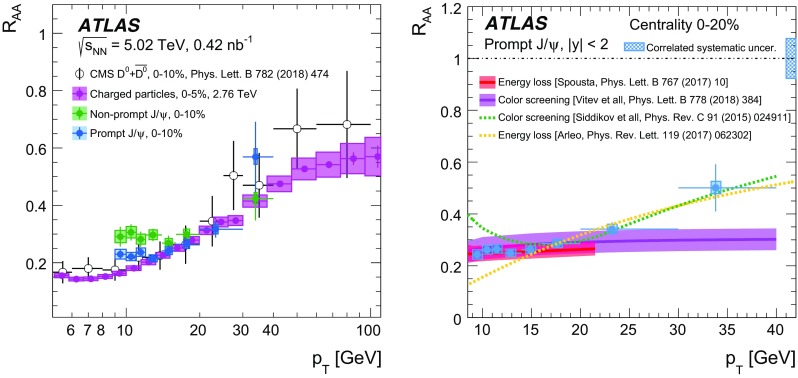
(Left) Comparison of prompt and non-prompt $J/\psi$ $R_{\text{ AA }}$ with the $R_{\text{ AA }}$ of charged particles [49] and D-mesons [51]. (Right) Comparison of the $R_{\text{ AA }}$ for prompt $J/\psi$ production with different theoretical models. The statistical uncertainty of each point is indicated by a narrow error bar. The error box plotted with each point represents the uncorrelated systematic uncertainty, while the shaded error box at $R_{\mathrm {AA}}$=1 represents correlated scale uncertainties

**Fig. 7:**
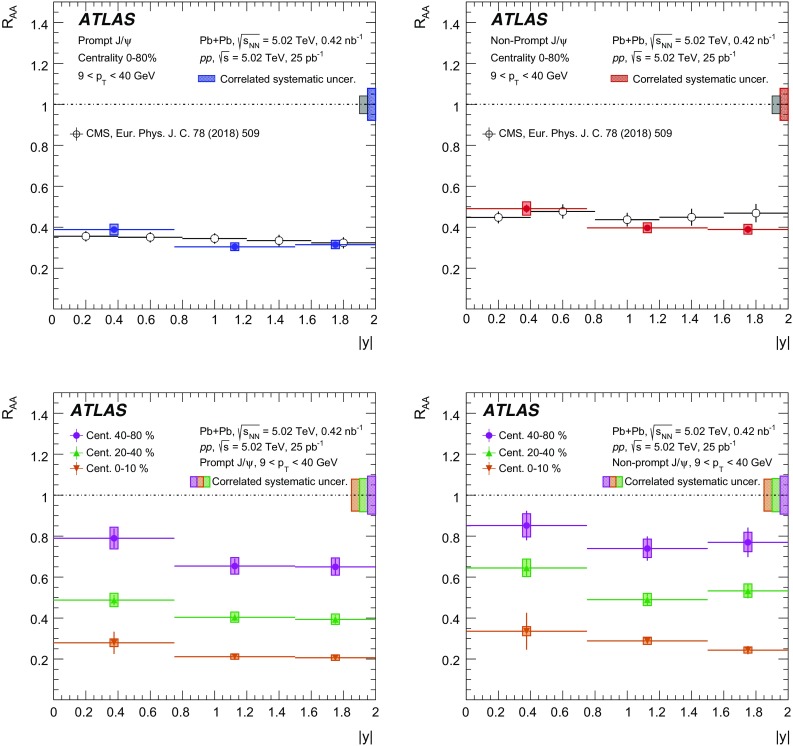
The nuclear modification factor as a function of rapidity for the prompt $J/\psi$ (left) and non-prompt $J/\psi$ (right) for $9< p_{\text {T}} < 40\ \text {GeV}$, in 0–80% centrality bin (top) and in 0–10%, 20–40%, and 40–80% centrality bins (bottom). The statistical uncertainty of each point is indicated by a narrow error bar. The error box plotted with each point represents the uncorrelated systematic uncertainty, while the shaded error box at $R_{\mathrm {AA}}$=1 represents correlated scale uncertainties

**Fig. 8:**
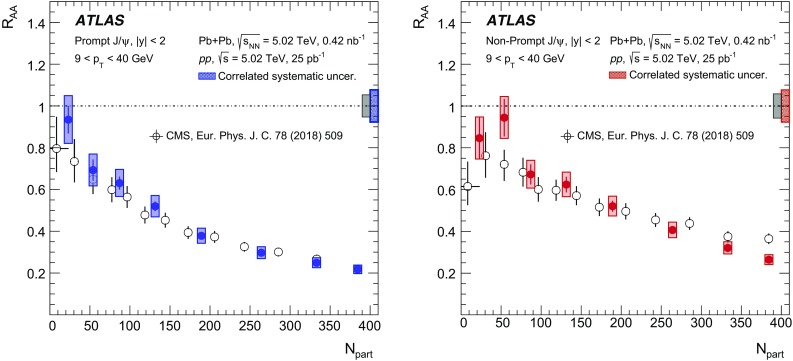
The nuclear modification factor as a function of the number of participants, $N_\mathrm {part}$, for the prompt $J/\psi$ (left) and non-prompt $J/\psi$ (right) for $9< p_{\text {T}} < 40\ \text {GeV}$ and for rapidity $|y|<2$. The statistical uncertainty of each point is indicated by a narrow error bar. The error box plotted with each point represents the uncorrelated systematic uncertainty, while the shaded error box at $R_{\mathrm {AA}}$=1 represents correlated scale uncertainties

The influence of the hot dense medium on the production of the $J/\psi$ mesons is quantified by the nuclear modification factor, given in Eq. (3), which compares production of charmonium states in Pb+Pb collisions to the same process in $pp$ collisions, taking geometric factors into account. The results of the measurement of this observable are presented as a function of transverse momentum in Figs. 5 and 6, rapidity in Fig. 7, and centrality in Fig. 8; the last is presented as a function of the mean number of participants. The error box on the right-hand side of the plots located at the $R_{\mathrm {AA}}$ value of 1 indicates the correlated systematic uncertainties of the measurement, while the error boxes associated with data-points represent the uncorrelated systematic uncertainties, and the error bars indicate the statistical uncertainties. The results exhibit agreement with previous measurements performed by CMS at $\sqrt{s_\text {NN}}= 2.76$ and 5.02 TeV in a similar kinematic region [11, 12], as can be seen in Figs. 5, 7 and 8 where the CMS results are plotted together with total uncertainties which are dominated by systematic uncertainties.

Figure 5 shows the nuclear modification factor as a function of $p_{\text {T}}$ for production of prompt and non-prompt $J/\psi$, for $|y| < 2$, and for four selections of centrality. In this figure, it can be seen that the production of $J/\psi$ is strongly suppressed in central Pb+Pb collisions. In the kinematic range plotted, as a function of $p_{\text {T}}$, the nuclear modification factor for both prompt and non-prompt $J/\psi$ production is seen to be in the range $0.2< R_{\mathrm {AA}} < 1$, depending on the centrality slice, having a minimum value for prompt $J/\psi$ of 0.229 ± 0.017(stat) ± 0.016(syst) and 0.290 ± 0.034(stat) ± 0.021(syst) for the non-prompt $J/\psi$ in the 0–10% centrality range. For $p_{\text {T}}$  > 12 GeV, a small increase in $R_\mathrm {AA}$ with increasing $p_{\text {T}}$ is observed in the prompt $J/\psi$ production, as shown in Fig. 6 (left), similar in shape and size to that observed for charged particles and *D*-mesons [49–51], typically attributed to parton energy-loss processes and, for the case of charmonia, also to coherent radiation from the pre-resonant $q\bar{q}$ pair [20, 21]. In Fig. 6 (right), one can see the prompt $J/\psi$ $R_{\text{ AA }}$ evaluated for the 0–20% centrality bin compared with several models, showing that the data are consistent with the colour screening and colour transparency picture [52–54], as well as parton energy-loss [20, 21]. The $R_{\text{ AA }}$ value for non-prompt $J/\psi$ is seen to be approximately constant as a function of $p_{\text {T}}$ within the uncertainties, also consistent with a parton energy-loss mechanism [55, 56].

In Fig. 7, the nuclear modification factor is presented as a function of rapidity for production of prompt and non-prompt $J/\psi$ for transverse momenta $9< p_\mathrm {T} < 40$ GeV and for four selections of centrality. It can be seen from the figure that the $R_\mathrm {AA}$ exhibits a modest dependence on rapidity, as expected from Ref. [57], explained due to the boost invariance of the medium in central rapidity region. These patterns are seen to be similar for both prompt and non-prompt $J/\psi$ production. Figure 8 presents the nuclear modification factor as a function of centrality, expressed as the number of participants, $N_\mathrm {part}$, for production of prompt and non-prompt $J/\psi$ for $|y| < 2$, and for $9< p_\mathrm {T} < 40$ GeV. In the kinematic range plotted, as a function of centrality, the nuclear modification factor for both prompt and non-prompt $J/\psi$ decrease from the most peripheral bin, 60–80%, to the most central bin, 0–5%, with a minimum value of 0.217 ± 0.010(stat) ± 0.020(syst) for prompt and 0.264 ± 0.017(stat) ± 0.023(syst) for non-prompt. Suppression by a factor of about 4 or 5 for both the prompt and non-prompt $J/\psi$ mesons in central collisions, together with $\mathrm {R}_\mathrm {pPb}$ of charmonia being consistent with unity [25], are a very striking signs that the hot dense medium has a strong influence on the particle production processes. The two classes of meson production have essentially the same pattern which is unexpected because the two cases are believed to have quite different physical origins: the non-prompt production should be dominated by *b*-quark processes that extend far outside the deconfined medium, whereas the prompt production happens predominantly within the medium.

**Fig. 9:**
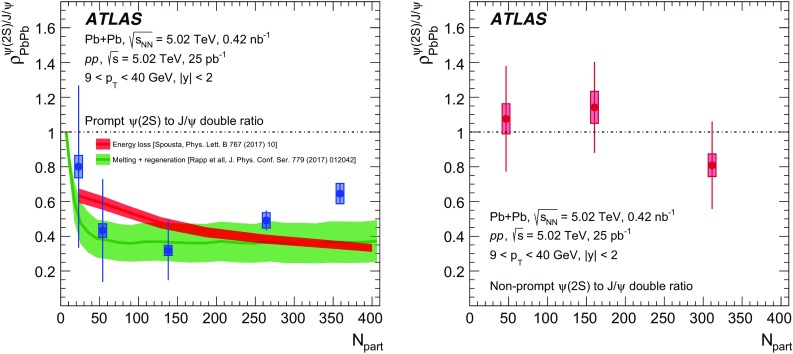
$\psi (2\mathrm {S})$ to $J/\psi$ double ratio, as a function of the number of participants, $N_\mathrm {part}$, for prompt meson production compared with different theoretical models (left) and non-prompt meson production (right). The narrow error bar represents the statistical uncertainties while the error box represents the total systematic uncertainty

### $\psi (2\mathrm {S})$ to $J/\psi$ yield double ratio

The double ratio of $\psi (2\mathrm {S})$ production to $J/\psi$ meson production, $\rho _\text {PbPb}^{\psi (2\mathrm {S})/J/\psi }$ is shown in Fig. 9 for the centrality bins of 0–10%, 10–20%, 20–50%, 50–60% and 60–80%. These results represent a measurement complementary to an earlier measurement of $\psi (2\mathrm {S})$ to $J/\psi$ yield ratios at the same centre-of-mass energy made by the CMS Collaboration [58]. This ratio, which compares the suppression of the two mesons, can be interpreted in models in which the binding energy of the two mesons is estimated [59], leading to different survival probabilities in the thermal medium, or in which the formation mechanisms differ, such as different susceptibility of the two mesons to recombination processes [60, 61]. If the non-prompt $J/\psi$ and $\psi (2\mathrm {S})$ originate from *b*-quarks losing energy in the medium and hadronizing outside of the medium, then the ratio of their yields should be unity. This statement should be true for the ratio expressed as a function of any kinematic variable. By contrast, prompt $J/\psi$ and $\psi (2\mathrm {S})$ or their pre-resonant states, should traverse the hot and dense medium. Considering both mesons as composite systems, with potentially different formation mechanisms and different binding energies, they may respond differently to the hot dense medium. This interpretation is supported by the results of Fig. 9, which shows the ratio of $\psi (2\mathrm {S})$ to $J/\psi$ production as a function of the number of collision participants, $N_\mathrm {part}$. The ratio is consistent with unity within the experimental uncertainties for non-prompt mesons, while for prompt $J/\psi$ the ratio is different from unity. These data support the enhanced suppression of prompt $\psi (2\mathrm {S})$ relative to $J/\psi$. This observation is consistent with the interpretation that the tightest bound quarkonium system, the $J/\psi$, survives the temperature of the hot and dense medium with a higher probability than the more loosely bound state, the $\psi (2\mathrm {S})$. It is, however, also consistent with the radiative energy-loss scenario as shown in Ref. [20]. Irrespective of the underlying mechanism for the charmonium suppression, one may expect less ambiguity in the interpretation of this result since quark recombination processes, $J/\psi$s formed from uncorrelated $c\bar{c}$ pairs in the plasma, which are important at small $p_\mathrm {T}^{\psi (\textit{n}\mathrm {S})}$, should not play a significant role here [17, 18, 62].

## Summary

Measurements of $J/\psi$ and $\psi (2\mathrm {S})$ production are performed in the dimuon decay channel in Pb+Pb collisions at $\sqrt{s_\mathrm {NN}}$ = 5.02 TeV with an integrated luminosity of $0.42~\mathrm {nb}^{-1}$, and in *pp* collisions at $\sqrt{s}$ = 5.02 TeV, with an integrated luminosity of $25~\text{ pb }^{-1}$ collected with the ATLAS experiment at the LHC. Results are presented for prompt and non-prompt nuclear modification factors of the $J/\psi$ mesons, as well as the yields and non-prompt fraction in the region with transverse momentum $9< p_{\text {T}} < 40\ \text {GeV}$ and rapidity $|y| < 2$.

Strong suppression of prompt and non-prompt $J/\psi$ and $\psi (2\mathrm {S})$ mesons is observed in Pb+Pb data. The maximum suppression of prompt and non-prompt $J/\psi$ is observed for the most central collisions. The dependence of the nuclear modification factor $R_{\mathrm {AA}}$ on centrality is approximately the same for prompt and non-prompt $J/\psi$. The prompt $J/\psi$ $R_\mathrm {AA}$, as a function of $p_{\text {T}}$, shows an increasing trend while the non-prompt $J/\psi$ $R_\mathrm {AA}$ is consistent with being constant as a function of $p_{\text {T}}$ within the uncertainties.

The ratio of $\psi (2\mathrm {S})$ to $J/\psi$ meson production is measured for both the prompt and non-prompt mesons, and is shown as a function of centrality. Values consistent with unity are measured for the non-prompt mesons, while the values observed for the prompt mesons are below unity.

## Footnotes

1 ATLAS uses a right-handed coordinate system with its origin at the nominal interaction point (IP) in the centre of the detector and the *z*-axis along the beam pipe. The *x*-axis points from the IP to the centre of the LHC ring, and the *y*-axis points upwards. Cylindrical coordinates $(r,\phi )$ are used in the transverse plane, $\phi$ being the azimuthal angle around the *z*-axis. The pseudorapidity is defined in terms of the polar angle $\theta$ as $\eta = -\ln \tan (\theta /2)$. Angular distance is measured in units of $\Delta R \equiv \sqrt{(\Delta \eta )^{2} + (\Delta \phi )^{2}}$.
